# Genomic and transcriptomic profiling reveals distinct molecular subsets associated with outcomes in mantle cell lymphoma

**DOI:** 10.1172/JCI153283

**Published:** 2022-02-01

**Authors:** Shuhua Yi, Yuting Yan, Meiling Jin, Supriyo Bhattacharya, Yi Wang, Yiming Wu, Lu Yang, Eva Gine, Guillem Clot, Lu Chen, Ying Yu, Dehui Zou, Jun Wang, An T. Phan, Rui Cui, Fei Li, Qi Sun, Qiongli Zhai, Tingyu Wang, Zhen Yu, Lanting Liu, Wei Liu, Rui Lyv, Weiwei Sui, Wenyang Huang, Wenjie Xiong, Huijun Wang, Chengwen Li, Zhijian Xiao, Mu Hao, Jianxiang Wang, Tao Cheng, Silvia Bea, Alex F. Herrera, Alexey Danilov, Elias Campo, Vu N. Ngo, Lugui Qiu, Lili Wang

**Affiliations:** 1 State Key Laboratory of Experimental Hematology, National Clinical Research Center for Blood Diseases, Haihe Laboratory of Cell Ecosystem, Institute of Hematology and Blood Diseases Hospital, Chinese Academy of Medical Sciences and Peking Union Medical College, Tianjin, China.; 2 Department of Systems Biology, Beckman Research Institute, City of Hope Comprehensive Cancer Center, Monrovia, California, USA.; 3 Division of Translational Bioinformatics, Beckman Research Institute, City of Hope Comprehensive Cancer Center, Irwindale, California, USA.; 4 Lymphoid Neoplasm Program, Institut d’Investigacions Biomèdiques August Pi i Sunyer (IDIBAPS), Hematology Department, Hospital Clínic, Departament d’Anatomia Patològica, Universitat de Barcelona, Barcelona, Spain.; 5 Toni Stephenson Lymphoma Center, Beckman Research Institute, City of Hope Comprehensive Cancer Center, Duarte, California, USA.; 6 Department of Hematology, Tianjin First Center Hospital, Tianjin, China.; 7 Department of Hematology, The First Affiliated Hospital of Nanchang University, Institute of Hematology, Academy of Clinical Medicine of Jiangxi Province, Nanchang, Jiangxi Province, China.; 8 Department of Pathology, National Clinical Research Center for Cancer, Key Laboratory of Cancer Prevention and Therapy of Tianjin, Tianjin Medical University Cancer Institute and Hospital, Tianjin, China.; 9 Department of Hematology and Hematopoietic Cell Transplantation, City of Hope Comprehensive Cancer Center, Duarte, California, USA.

**Keywords:** Genetics, Oncology, Genetic variation, Lymphomas

## Abstract

Mantle cell lymphoma (MCL) is a phenotypically and genetically heterogeneous malignancy in which the genetic alterations determining clinical indications are not fully understood. Here, we performed a comprehensive whole-exome sequencing analysis of 152 primary samples derived from 134 MCL patients, including longitudinal samples from 16 patients and matched RNA-Seq data from 48 samples. We classified MCL into 4 robust clusters (C1–C4). C1 featured mutated immunoglobulin heavy variable (IGHV), *CCND1* mutation, amp(11q13), and active B cell receptor (BCR) signaling. C2 was enriched with del(11q)/*ATM* mutations and upregulation of NF-**κ**B and DNA repair pathways. C3 was characterized by mutations in *SP140*, *NOTCH1*, and *NSD2*, with downregulation of BCR signaling and MYC targets. C4 harbored del(17p)/*TP53* mutations, del(13q), and del(9p), and active MYC pathway and hyperproliferation signatures. Patients in these 4 clusters had distinct outcomes (5-year overall survival [OS] rates for C1–C4 were 100%, 56.7%, 48.7%, and 14.2%, respectively). We also inferred the temporal order of genetic events and studied clonal evolution of 16 patients before treatment and at progression/relapse. Eleven of these samples showed drastic clonal evolution that was associated with inferior survival, while the other samples showed modest or no evolution. Our study thus identifies genetic subsets that clinically define this malignancy and delineates clonal evolution patterns and their impact on clinical outcomes.

## Graphical abstract

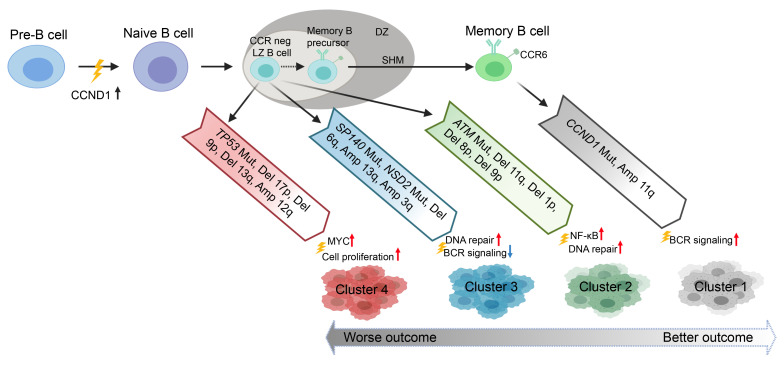

## Introduction

Mantle cell lymphoma (MCL) is an aggressive subtype of non-Hodgkin’s B cell lymphoma that has a median overall survival (OS) of approximately 5 years (1–7). MCL can generally be grouped into 2 types based on clinical indications: aggressive conventional MCL (cMCL) and indolent leukemic nonnodal MCL (nnMCL) (2, 5). Several clinical and molecular features are used to distinguish these 2 types of MCL, including involvement of lymph nodes, expression of SOX11 (2, 5), and somatic hypermutation on the B cell receptor (BCR) immunoglobulin heavy variable (IGHV) genes (1, 5, 8, 9). Due to significant heterogeneity in the clinical outcome of patients with MCL (3, 4, 10), differentiating patients who will have poor clinical outcomes from patients who will achieve durable response with standard therapies remains a challenge. The MCL International Prognostic Index (MIPI; refs. 7, 11–14) and tumor Ki67 (11) expression are used to stratify newly diagnosed MCL patients. However, these traditional prognostic markers have not enabled tailored therapeutic strategies for MCL. In the era of novel therapies for MCL (1, 15, 16), better prognostic and predictive models that account for the biologic heterogeneity of the disease are needed to stratify patients.

In the last decade, unbiased massively parallel sequencing of whole exomes (WES) and RNA-Seq of MCL have identified recurrent mutations (*TP53*, *ATM*, *NOTCH1/2*, *CCND1*, *HNRNPH1*, *KMT2D*) associated with MCL (6, 17–22) and genetic lesions (del[9p], *ARID1A*, *SMARCA4*) that contribute to resistance to chemoimmunotherapy or targeted therapies (21, 23–25). However, several constraints have limited previous analyses. First, use of relatively small cohorts in studies that utilized an unbiased discovery approach (17, 21, 26) has curtailed the ability to define patterns of genetic lesions and their associations with clinical outcomes. In a larger study (25), only 8 genes were assessed, allowing limited evaluation of the prognostic importance of cooccurring genetic alterations. Second, limited availability of matched RNA-Seq and WES data impeded connecting the genotype with phenotype. Finally, lack of longitudinal samples restricted the ability to evaluate clonal evolution of MCL in relation of disease progression to chemoimmunotherapy.

To address these limitations, we performed WES on 152 MCL tumor samples from 134 patients (Table 1). Longitudinally collected samples were available for 16 patients, and 48 samples had matched RNA-Seq data (Supplemental Figure 1; supplemental material available online with this article; https://doi.org/10.1172/JCI153283DS1).

## Results

### Mutated cancer driver genes and mutational signatures in MCL.

Our samples were collected from 134 MCL patients (123 newly diagnosed and 11 with relapsed disease, 15 nnMCL and 119 cMCL) with a broad range of clinical characteristics, including different MIPI risk as well as IGHV unmutated and mutated (Table 1 and Supplemental Tables 1 and 2). The median follow-up time was 31.0 (range 4.5–107.3) months. The 3-year OS rate in the cohort was 69.6% (95 CI, 60.1%–78.5%).

**Figure 1:**
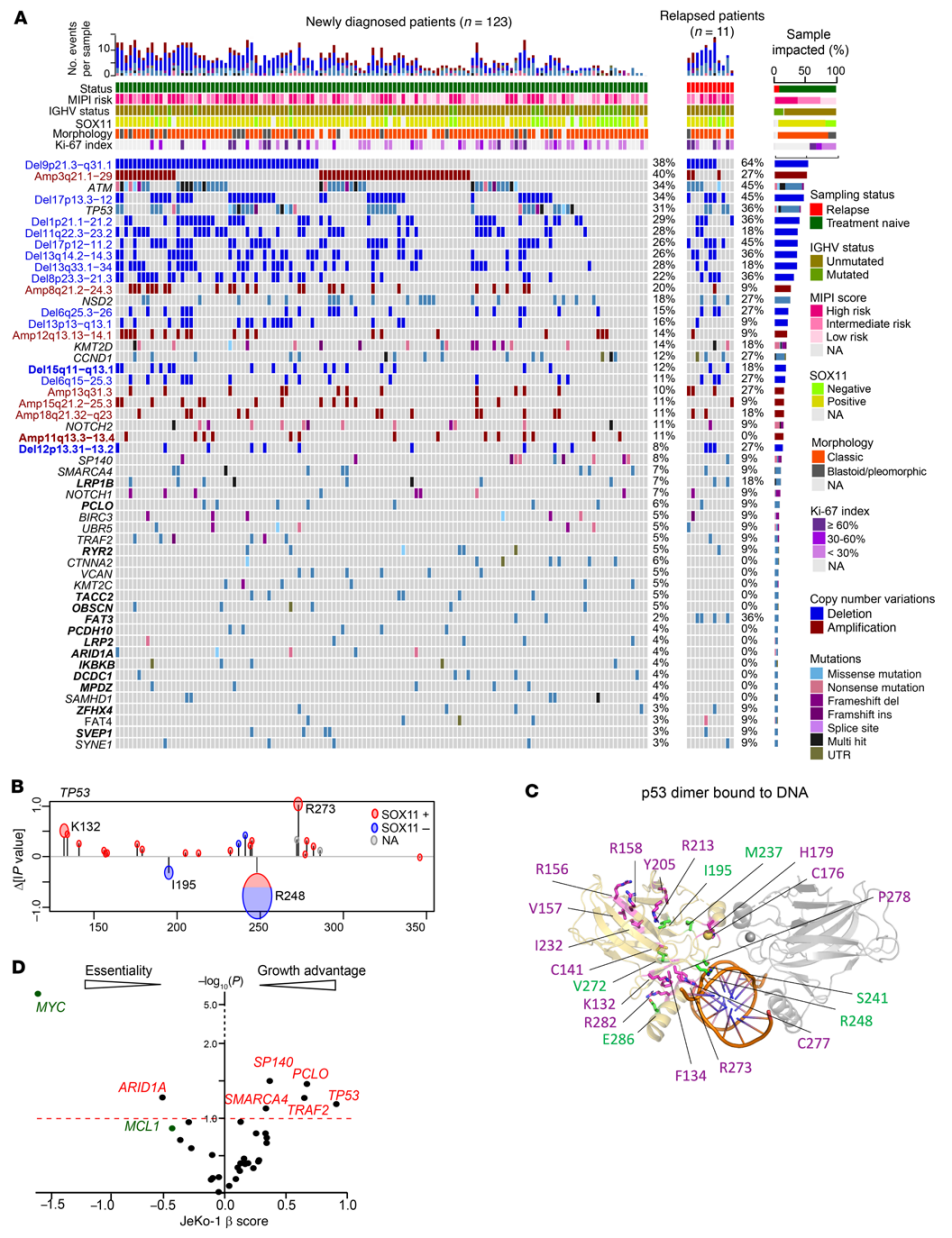
Recurrent somatic genetic alterations and mutation signatures in MCL. (**A**) Recurrent somatic mutations and CN alterations (rows) identified following WES of 134 primary samples (columns) obtained from patients with newly diagnosed (green) and relapsed (red) MCL. Samples were annotated for prior treatment, MIPI risk, IGHV status, and Sox11 expression level when collected. Left: blue labels, recurrent CN deletion; red labels, recurrent CN amplification; black labels, somatic mutations; bold labels, novel CN alterations/mutations. Right: percentage of samples mutated. Top: total number of genetic alterations across the cohort. (**B**) Contributions of individual mutations to the collective WAP score of TP53. The changes in WAP score *P* value due to removal of individual mutations are plotted as function of residue number. The radius of the circles around each point in the graphs represent the number of patients with that mutation. Color indicates *SOX11* expression. (**C**) TP53 dimer bound to DNA fragment, PDB ID: 3IGK. One of the monomers is shown in yellow, the other in gray. DNA is shown in orange. The mutations observed in SOX11^+^ and SOX11^–^ patients are shown as magenta and green, respectively. (**D**) β Scores from genome-wide CRISPR/Cas9 screens of JeKo-1 of genes identified as having recurrent mutations.

We detected a median of 29 nonsynonymous mutations in protein-coding sequences per sample (range, 8–72), and a median mutation burden of 1.35 mutations per megabase (Mb), which is similar to that previously reported in MCL and other hematologic cancers (Supplemental Figure 3, A and B, and refs. 17, 19, 21, 26). We identified 33 recurrently mutated genes (mutated in >5 samples, mutation frequency >3%, Figure 1A), which included known and novel recurrent mutations (*LRP1B*, *PCLO*, *RYR2*, *PCDH10*, *OBSCN*, *TACC2*, *FAT3*, *LRP2*, *SVEP1*, *ZFHX4*, *MPDZ*, *DCDC1*, *IKBKB*, *ARID1A*; Figure 1A).

To determine which of the mutated genes are likely to contribute to lymphomagenesis, we used the clustering of mutations in protein structures (CLUMPS) algorithm (27) to identify clustering of mutations predicted to have significant impact on 3D protein structures or interference with protein’s binding partners. Mutations in *TP53* and *CCND1* were found with significant clustering (*P* < 0.05), whereas mutations in *ATM*, *SP140*, and *SMARCA4* showed moderate clustering (Supplemental Figure 4A; *P* < 0.1). To determine how individual mutation affects the clustering *P* value, we systematically removed each mutation and recalculated the weighted average proximity (WAP) score and the resulting change in –log_10_ (*P* value), Δlpvalue (Figure 1B and Supplemental Figure 4C). A positive Δlpvalue indicates that the mutation clusters with other mutations in the protein and that the removal of this mutation adversely affects the CLUMPS score significance. Conversely, a negative Δlpvalue indicates that the mutation does not cluster significantly with other mutations. In TP53, we found both categories of mutations with positive and negative Δlpvalues (Figure 1C). For example, mutations at R248 and I195 showed negative Δlpvalues, while mutations at R273 had positive Δlpvalues, indicating that these 2 groups of *TP53* mutations may exert their effects in different ways. Two of the mutations with negative Δlpvalue, R248 and S241, were at the DNA-binding interface, suggesting that these mutations may interfere with the DNA recognition by TP53. In contrast, mutations with positive Δlpvalues, such as R158, V156, and Y205, were clustered within structural domains of TP53 that were distant from the DNA-binding interface. These mutations may affect the function of TP53 through different mechanisms compared with the ones that are unclustered. We further observed that the Δlpvalue of the WAPscore for the *TP53* mutation was significantly different between SOX11-negative and -positive patients (Figure 1B and Supplemental Figure 4B). This implies that the SOX11-positive patients tend to have mutations that cluster together in the TP53 structure compared with SOX11-negative patients (Figure 1C). The hotspot mutations at the C47 and Y44 of CCND1 affecting the weighted average proximity (WAP) score were not in direct contact with its binding partner CDK4, but in a loop region that packs between 2 helices and maintains a half helical turn in the loop (Supplemental Figure 4E). The significance was supported by the observation that both mutations can increase CCND1 protein stability and promote ibrutinib resistance in MCL (28).

To delineate the roles of recurrent mutations in MCL biology, we examined genome-wide CRISPR/Cas9 perturbation screen results from DepMap (https://depmap.org/portal/) for leukemia and lymphoma as well as our own CRISPR/Cas9 perturbation screen results in the MCL cell line JeKo-1. Silencing of *SP140*, *SMARCA4*, *PCLO*, *TP53*, and *TRAF2* in JeKo-1 cells conferred a cell-growth advantage, while knockdown of these genes in other cell lines had modest or no impact on cell growth (Figure 1D and Supplemental Figure 3C), indicating that these genes may act as tumor suppressors in MCL.

Leveraging our WES data set, we identified 4 mutation signatures prevalent in MCL using the MutationalPatterns pipeline (Supplemental Figure 5, A and B,and ref. 29). This includes an age-related signature involving C-to-T transitions at CpG sites; a c-AID signature characterized by increased C>T/G mutations at a known activation-induced cytidine deaminase (AID) hotspot (SBS84); an enzyme essential for somatic hypermutation of germinal center B cells; and signatures 5 and 40, common signatures that were prevalent in most cancers and leukemia/lymphoma, respectively. The c-AID signature mainly comprised clustered mutations (Supplemental Figure 5C). Of note, most of the signatures contributed by aging-related signature and signature 40 (range: 36.2%–100%, median 68.8%, Supplemental Figure 5D).

### Copy number alterations in MCL.

**Figure 2:**
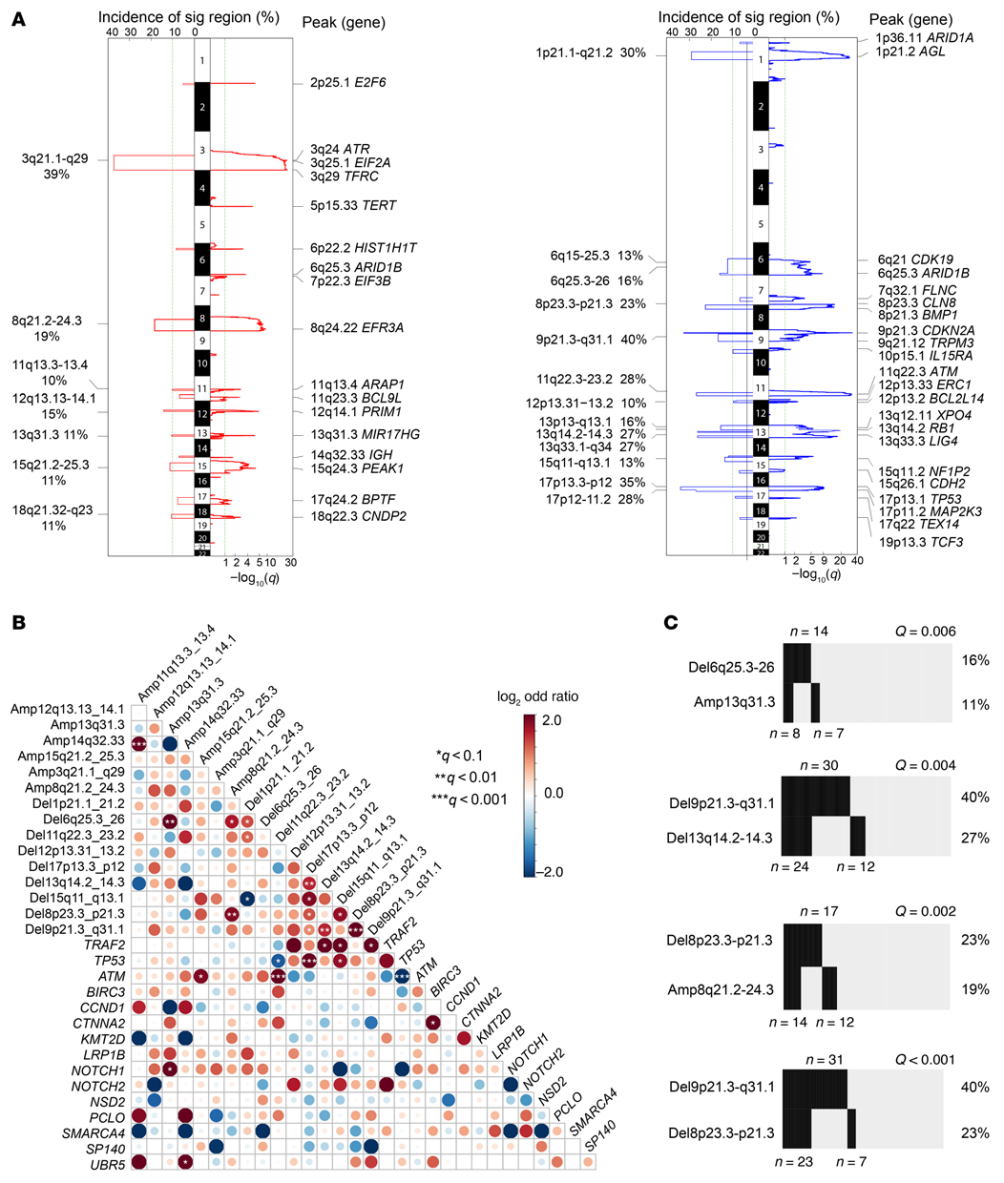
Recurrent SCNAs, cooccurring genetic events, and clinical association. (**A**) Significant CN amplifications (left, red) and deletions (right, blue). Left sides of the mirror plots show the incidence of significant focal CNA events. Right sides of the mirror plots show *q* values for each region. Genes located in the peak of relevant cytobands are listed. (**B**) Pairwise associations between recurrent genetic alterations found in the 134 MCL samples. Low and high cooccurrence are shown in blue and red, respectively. Intensity of the color reflects the odds ratio. Statistically significant association as determined by *q* value is marked by asterisks. (**C**) Number of samples with cooccurrence of the indicated genetic events in the cohort of 134 MCL samples. *S*ignificance of Fisher’s exact test indicated by *q*.

With this data set, we identified 20 recurrent somatic copy number alterations (SCNAs) (Figure 1A and Figure 2A; *q* value ≤ 0.1, frequency ≥ 10%). Of note, the tumor-only pipeline generated highly correlated SCNA calls in the 89 paired samples, which were well correlated with FISH results (receiver operating characteristic [ROC], *P* < 0.001 for del[17p], del[13q], del[11q]) (Supplemental Figure 6, A and B, and Supplemental Figure 7). In addition to previously reported SCNAs linked to OS (del[9p], del[17p], del[13q], and del(8p23.3]; refs. 18, 30), we also identified driver SCNAs, including del(15q11–13) and amp[11q13.3) (Figure 1A).

To determine how SCNAs affect gene expression, we performed an integrative analysis in samples with WES and RNA-Seq data (*n* = 48). We focused on identifying genes that showed significant changes within the deleted or amplified regions by comparing samples with or without the lesions. Pathways that were significantly affected by amplification included RNA catabolic and translation pathways (*EIF4G1*, *RPL4*, *DDX6*, *PRL15*) and the MYC pathway (*MYC*, *NME1*). Pathways that were perturbed by deletion included DNA repair and cell cycle (*ATM*, *CDKN1B*, *POT1*) and RNA splicing (*HNRNPK*, *NCBP1*, *SRSF1*) (Supplemental Figure 6, C and D).

Our WES data set revealed significant relationships between several genetic alterations (Figure 2B). In addition to known cooccurrence between *TP53* mutation and del(17p), *ATM* mutation and del(11q), del(9p21.3) and del(17p) (Supplemental Figure 8, A–C), we also observed a high cooccurrence of del(9p21.3) with del(8p) (*q* < 0.001) and del(13q) (*q* = 0.004; Figure 2C). Moreover, we found low cooccurrence of genetic alterations such as mutations in *TP53* and *ATM* or del(11q) (Supplemental Figure 8B; *q* < 0.05), indicating tumor cells harboring these events may originate from a different genetic trajectory.

### Association of genetic features with clinical outcomes.

We examined associations between genetic alterations and key MCL features. Overall, we observed a high number of SCNAs associated with unmutated IGHV status and SOX11 expression (Supplemental Figure 9, A and B). The c-AID mutation signature was strongly associated with mutated IGHV status, while the aging signature correlated with unmutated IGHV and SOX11 expression (Supplemental Figure 9, C and D). Moreover, the number of SCNAs was able to predict clinical outcomes (Supplemental Figure 9, E and F).

**Figure 3:**
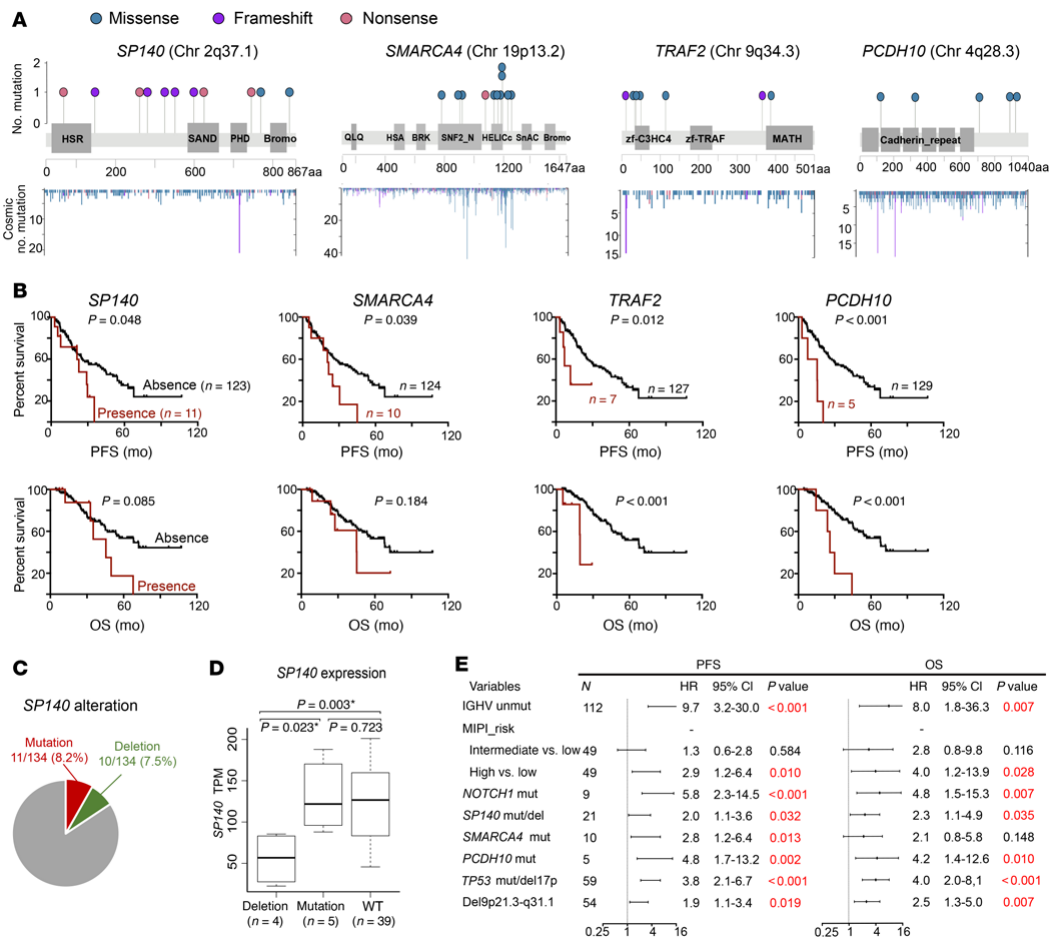
Associations of somatic mutations with clinical outcomes. (**A**) Lollipop diagrams of selected putative driver genes showing mutation subtype, position, and frequency. Bottom: *y* axis indicates the number of identified mutations in the COSMIC database. (**B**) Kaplan-Meier plots (with log-rank *P* values) of PFS and OS associated with presence and absence of selected mutations. (**C**) Samples with *SP140* mutations or deletions did not overlap in the cohort. (**D**) Deletion of *SP140* affected its gene expression. *SP140* expression TPM value was extracted and plotted from MCL samples with *SP140* deletion, mutation, or WT. *P < 0.05. (**E**) Forest plots of the multivariate analysis of MIPI risk groups and individual genetic factors for PFS and OS in our MCL cohort.

We examined the prognostic significance of somatic mutations. Mutations in *SP140*, *SMARCA4*, *TRAF2*, and *PCDH10* were predictive of poor progression-free survival (PFS) (Figure 3, A and B). *SP140* mutations occurred at 8% frequency in our cohort, and 9 out of 11 mutations were frameshift and nonsense mutations that resulted in a truncated form of *SP140* (Figure 3A), highly suggestive of loss-of-function mutations. We further identified 10 samples (7.5%) harboring *SP140* deletion (loss of 2q36.3–37.1), all of which showed downregulation of *SP140* expression compared with samples lacking the deletion (Figure 3, C and D). Mutation or deletion of *SP140* was predictive for shorter PFS and OS and associated with SOX11 expression, suggesting this gene may be a potential tumor suppressor in MCL (Supplemental Figure 9G and Supplemental Figure 10A). Consistent with previous publications (25, 31), the presence of *TP53* or *NOTCH1* aberrations was associated with shorter PFS (Supplemental Figure 10, B and C, and Figure 3E). Of note, *TP53*, *NOTCH1*, and *PCDH10* mutations as well as the *SP140* mutation/deletion retained significance for PFS and OS when MIPI risk and IGHV mutation status were added (Figure 3E).

We then assessed the contribution of recurrent SCNAs to MCL progression. Consistent with previous observations (30, 32–35), loss of 17p13.3 (35%) and 9p21.3 (40%) predicted inferior PFS and OS (Supplemental Figure 11), and this remained significant in the multivariate analysis (Figure 3E). Recurrent SCNAs in this cohort including del(12p13.31), del(13q14.2), del(15q11–13), del(8p23.3), and amp(13q31) were also associated with shortened PFS and OS (Supplemental Figure 11), but this was not significant in multivariable analysis (Figure 3E). Del(9p21.3), del(1p21.1), del(11q22.3), del(13q14.2), and del(6q25.3) were associated with unmutated IGHV and SOX11 expression (*P* < 0.05; Supplemental Figure 9G). Most of the genetic alterations also remained significant among patients who received the cytarabine-based regimen (Supplemental Figure 10, D and E).

**Figure 4:**
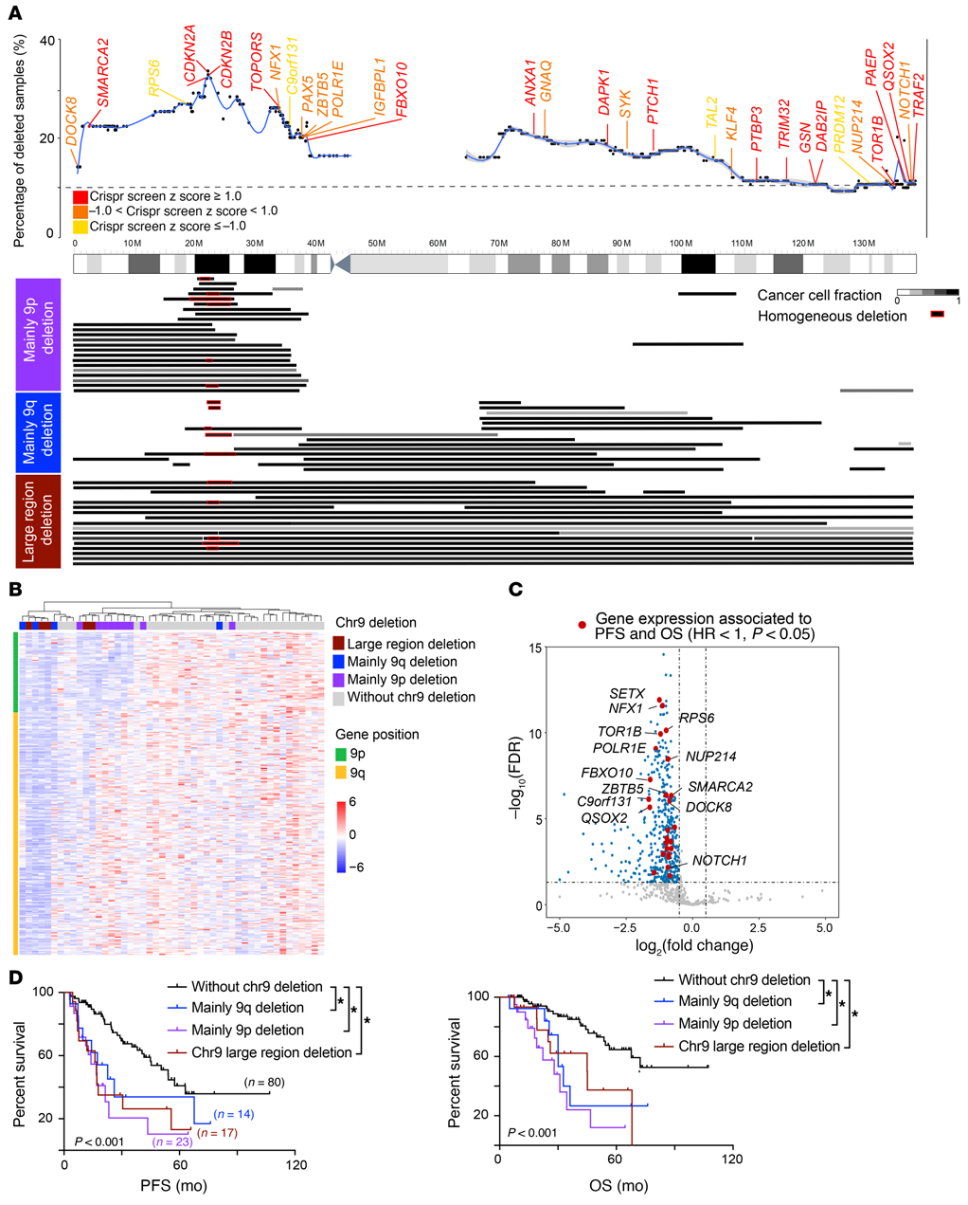
Deletion of chromosome 9 was associated with poor survival. (**A**) Chromosome 9 deletion in samples from our cohort. Top: blue line indicates percentage of MCL samples with chromosome 9 deletion at the location. Known tumor suppressors and oncogenes present on chromosome 9 are color coded based on their *z* score in the CRISPR/Cas9 screen in JeKo-1 cells. Bottom: deletions in 9p (purple), 9q (blue), or large regions (dark red) in samples from our cohort. Homozygous minimal 9p deletions are marked in red. CCF (Supplemental Methods) of chromosome 9 deletion is shown in gray scale. (**B**) Unsupervised clustering analysis of gene expression in chromosome 9 distinguishes MCL samples with deletions in different region. (**C**) Volcano plot of genes on chromosome 9 that are differentially expressed between MCL samples that have and do not have chromosome 9 deletions. Downregulated genes that were significantly associated with shorter PFS and OS are indicated in red (Cox’s regression HR <1, *P* < 0.05). (**D**) Kaplan-Meier plots of PFS and OS according to type of chromosome 9 deletion.

We further gained insights into the contribution of the most frequent deletion in MCL, the chromosome 9 deletion. We first examined genes that may render cell-growth advantage through analysis of our CRISPR/Cas9 perturbation screen in JeKo-1 cells and found that many critical tumor suppressors were located on chr9, including *CDKN2A*, *SMARCA2*, *FBXO10*, and *TOR1B* (Figure 4A; *z* score ≥ 1). We next classified the WES samples with del(9) into 3 groups based on the deleted region: 9p^–^, 9q^–^, or large region (both 9p/9q) (Figure 4A). Del(9p) was more frequent (23/54) than del(9q) (14/54) or both (17/54; Figure 4A). These deletions also influenced gene expression as reflected by our unsupervised RNA-Seq analysis of MCL samples containing and lacking these deleted regions (Figure 4B). Consistent with a previous study (36), we found that 24 downregulated genes on chr9 were significantly associated with PFS and OS (Figure 4C; HR < 1; *P* < 0.05), and all 3 types of deleted regions were predictive of inferior clinical outcomes (Figure 4D, log rank paired comparison, *P* < 0.05) irrespective of the size and location of the deleted region.

### Coordinate genetic signatures classify MCL into 4 subsets that have unique gene expression patterns and distinct clinical behavior.

**Figure 5:**
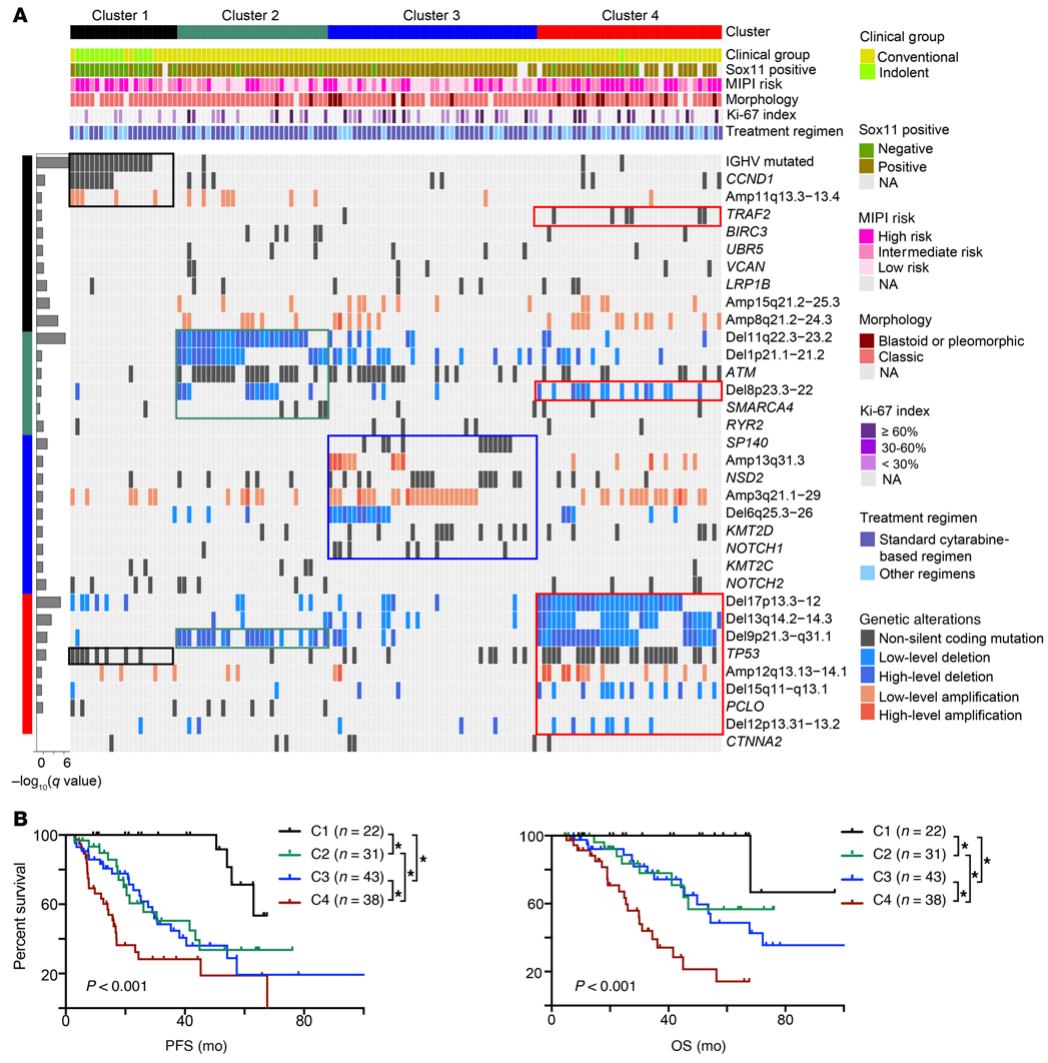
Coordinate genetic signatures group MCL into 4 clusters associated with clinical outcome. (**A**) Nonnegative matrix factorization (NMF) consensus clustering was performed using all somatic mutations and SCNAs in the 134 MCL samples (columns). Clusters 1 to 4 are shown with their associated landmark genetic alterations (boxed for each cluster). Left bar graph shows the correlation of genetic alterations associated with each cluster (*q* value, Fisher’s exact test). Nonsynonymous mutations, black; low-level deletion (1.0 ≤ CN ≤ 1.7 copies), light blue; high-level deletion (CN ≤ 1.0 copies), dark blue; low-level amplification (3.7 ≥ CN ≥ 2.3 copies), orange; high-level amplification (CN ≥ 3.7 copies), red. Header shows cluster association (C1, black; C2, green; C3, blue; C4, red), clinical group (cMCL, yellow green; nnMCL, light green), Sox11 expression (negative, green; positive, brown), MIPI risk (high risk, dark pink; intermediate risk, median pink; low risk, light pink), pathology status (blastoid or pleomorphic, crimson; classic, bright lilac), and treatment regimen (standard cytarabine-based aggressive regimen, dark blue; other regimen, light blue). (**B**) Kaplan-Meier plots of PFS and OS of patients grouped into the 4 clusters. *P < 0.05, log-rank test.

To identify genetic subtypes based on shared genetic features in MCL, we applied a nonnegative matrix factorization (NMF) consensus clustering algorithm (37, 38) to 35 recurrent genetic alterations and discovered 4 robust subsets of tumors characterized by distinct genetic signatures (Supplemental Figure 12A and Figure 5A). The 4 subtypes differed significantly in PFS and OS (Figure 5B; *P* < 0.001). Patients with the C1 subtype had a more favorable outcome than those with C2, C3, and C4 subtypes. Median PFS was not reached for C1 and was 41.2 months for C2, 30.7 months for C3, and 16.1 months for C4 (log rank, *P* < 0.001). Five-year OS rates for C1–C4 were 100%, 56.7%, 48.7%, and 14.2%, respectively. Differences in survival of patients of the 4 subtypes also remained significant among patients who received the cytarabine-based regimen (Supplemental Figure 12B). Moreover, molecular cluster was an independent risk factor when MIPI risk and IGHV mutation status were included in the multivariate analysis; however, this was mainly driven by C4 and C1 (Supplemental Figure 12C; C4 vs. C1, *P* = 0.017).

**Figure 6:**
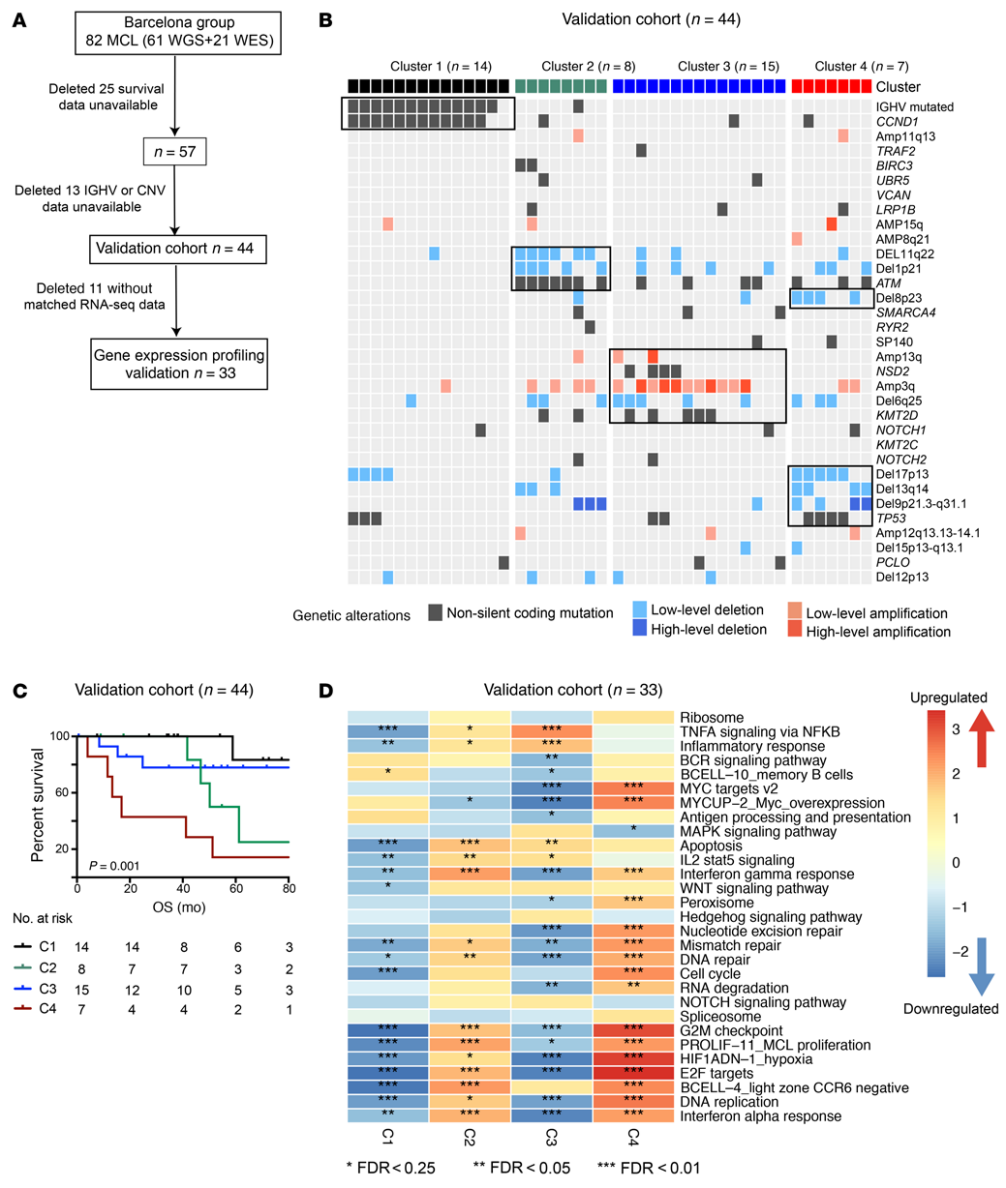
Molecular cluster and gene expression signature validated in Barcelona cohort. (**A**) Sample inclusion description in the validation cohort. (**B**) Projective nonnegative matrix factorization consensus clustering was performed using genetic alterations identified from our discovery cohort (Figure 5A). Clusters 1–4 are shown with their associated landmark genetic alterations (boxed for each cluster). Header shows cluster association (C1, black; C2, green; C3, blue; C4, red). (**C**) Kaplan-Meier plots of OS of patients grouped into the 4 clusters. *P* indicates log-rank test. Number indicates samples included in each cluster. (**D**) Integration of genetic and transcriptomic analyses identified gene expression signatures for each genetic subset. The heatmap was generated using normalized enrichment score (NES). Asterisks indicate the significance level of the enrichment.

To determine the robustness of these genetic clusters, we assessed whether these genetic alterations can stratify MCL patients using a published genetically well-annotated MCL cohort for validation (26) (Barcelona cohort, Figure 6, A–D). Projection of cluster features classified patients into 4 distinct clusters, with C1 having a favorable clinical course and C2 and C3 falling in between C1 and C4. There was significant statistical difference among clusters (*P* = 0.001), in which C1 versus C4 (*P* < 0.001), C2 versus C4 (*P* < 0.035), and C3 versus C4 (*P* = 0.014) reached significance in the pairwise test.

**Figure 7:**
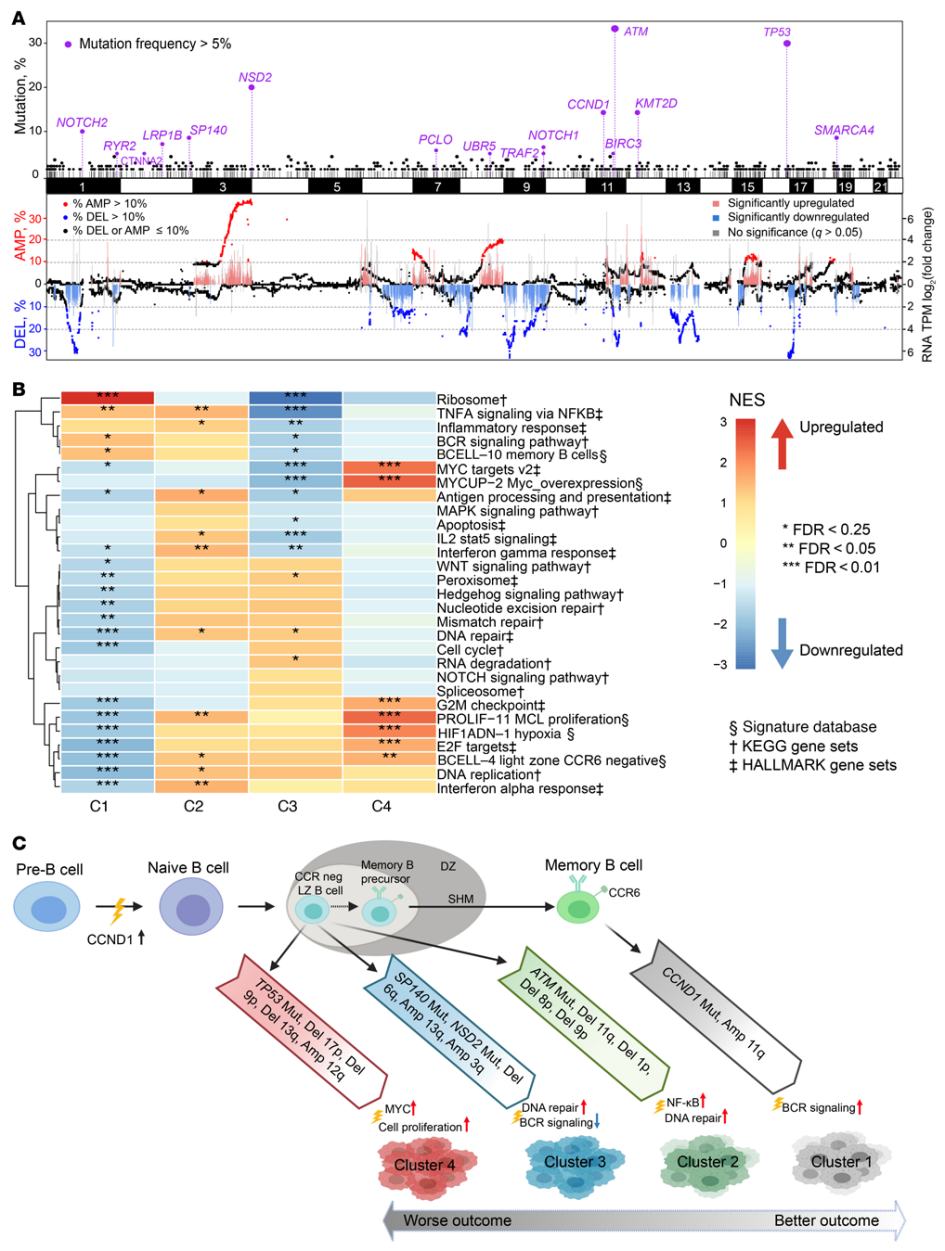
Integrative analysis of genome and transcriptome reveals a unique gene expression signature in each cluster. (**A**) Recurrent somatic mutations, SCNAs, and gene expression associated with SCNAs. Top panel: *x* axis shows the chromosome location of recurrent somatic mutations; *y* axis indicates the frequency of mutations detected in our MCL cohort (*n* = 134). Genes shown in purple have a mutation incidence of greater than 5%. Bottom panel: left *y* axis indicates proportions of CN deletion (DEL) and amplification (AMP). Each dot represents a gene at its chromosome location. Genes with absolute CN < 1.7 or > 2.3 were defined as deleted or amplified, respectively. Genes with a deletion incidence > 10% are shown in blue, and genes with an amplification incidence > 10% as red. (**B**) Integration of genetic and transcriptomic analyses identified unique gene expression signatures for each genetic subset. The Hallmark and KEGG gene sets and Signature database were used for Gene Set Enrichment Analysis. The heatmap was generated using normalized enrichment score (NES). Red indicates an upregulated pathway in the cluster compared with other clusters, while blue indicates a downregulated pathway. Asterisks indicate the significance level of the enrichment. (**C**) Proposed model for the 4 MCL subgroups. Clusters 1–4 were all associated with distinct genetic events and gene expression signatures. C1 had indolent disease and carried memory B cell gene signature. C2–C4 had more aggressive clinical courses and expressed CCR6-negative light zone or naive B cell gene signature.

To explore phenotypic differences among the MCL genetic subtypes, we performed an integrative analysis using matched RNA-Seq data (*n* = 48) across the 4 subsets (Figure 7, C1–C4, *n* = 12, 11, 16, and 9, respectively). We first assessed whether the recurrent mutated genes identified from WES were expressed at the RNA level and discovered most of these mutations were highly expressed (Supplemental Figure 13, A and B). Likewise, the frequent SCNAs also resulted in significant dysregulated gene expression (Figure 7A), which we further validated by reverse transcriptase PCR (RT-PCR) analysis of MCL samples containing and lacking the SCNAs (Supplemental Figure 13C). Our analysis revealed that each genetic subset has a unique gene expression pattern (Figure 7B and Supplemental Figure 14A). Consistent with the differing cellular origins for the 2 types of MCLs (1, 5), C1 was enriched for gene expression signatures of memory B cells and C2, C3, and C4 appeared to have a signature of CCR6-negative light zone B cells or naive B cells (Figure 7, B and C, and Supplemental Figure 14A). We further tested a previous reported 16-gene signature that distinguished cMCL and nnMCL (39) and found that 35 out of 36 C2–C4 patients were classified as cMCL while 9 out of 12 C1 patients were classified as nnMCL (Supplemental Figure 14B).

### C1.

The C1 group included 16% of samples. Most of the C1 samples were IGHV mutated and featured mutant *CCND1*, *TP53*, and amp(11q13). Most of the *TP53* mutations in C1 had negative WAP scores (Supplemental Figure 4B). Patients with and without *TP53* mutations had similar OS (*P* = 0.470, Supplemental Figure 15, A and B). Those in the C1 group had the lowest SOX11 expression (Supplemental Figure 15C). Phenotypically, C1 was enriched with a memory B cell phenotype and active BCR signaling (Figure 5A, Figure 7, B and C, and Supplemental Figure 14A). We observed enrichment of BCR signaling in the Barcelona cohort, although it was insignificant due to the small amount of available microarray gene expression data (Figure 6D).

### C2.

The C2 group included 23% of samples. Of 31 samples in C2, 28 harbored del(11q) (minimal deleted region contains *ATM*), while 19 of these 28 samples had a cooccurring *ATM* mutation. Consistent with these genetic lesions, genes involved in DNA replication, DNA repair, and hyperproliferation were all upregulated (Figure 7B). Expression of genes involved in TNF-α signaling via the NF-κB pathway and IFN-α and IFN-γ response was significantly enriched in both discovery and validation cohorts (Figure 6D and Figure 7B).

### C3.

The C3 group included 32% of samples. Besides enriched *NOTCH1* mutations, the C3 group also harbored mutations in *NSD2* *(WHSC1)*, *KMT2D*, and *SP140* as well as amp(13q) and del(6q). In contrast with C2, we observed significant downregulation of genes implicated in TNF-α signaling via the NF-κB pathway and IFN-γ response, but with activated NOTCH signaling. Additionally, BCR signaling, MYC targets, and IL-2 STAT5 signaling were all downregulated in C3 in both discovery and validation cohorts (Figure 6D and Figure 7, B and C).

### C4.

The C4 group included 28% of samples. This subtype harbored the most SCNAs, including deletions del(17p), del(13q), and del(9p) and mutations *TP53* and *TRAF2* (Figure 5A). Mutations in *TP53* were enriched for positive WAP score and predicted for poor survival in C4 (Supplemental Figure 15A). Phenotypically, C4 had gene signatures of the active MYC pathway, hyperproliferation, and light zone CCR6-negative B cells in both discovery and validation cohorts (Figure 6D and Figure 7, B and C). C4 was associated with the highest incidence of blastoid or pleomorphic MCL (25.0%, *P* = 0.016) and SCNAs (*P* < 0.001; Supplemental Figure 15, C and D), but had the lowest contribution to the clustered cAID mutation signature (*P* < 0.001; Supplemental Figure 15E). Consistent with this, C4 had the worst clinical outcome, with median PFS and OS of 16.1 and 30.0 months, respectively.

### Temporal ordering of genetic events and clonal evolution during progression of MCL.

**Figure 8:**
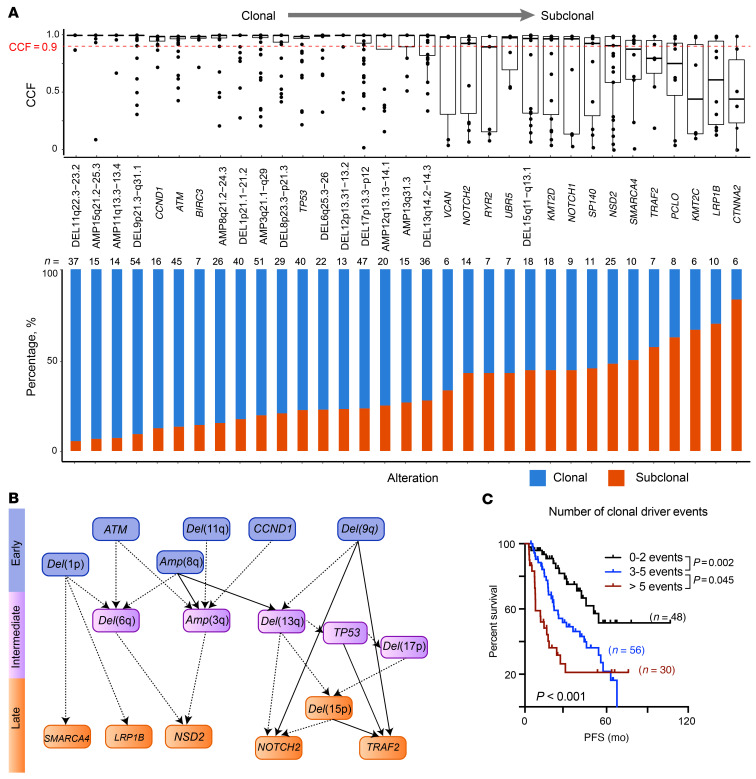
Clonal driver events associated with clinical outcomes. (**A**) CCF values for each sample affected by a recurrent genetic alteration across all 134 samples. Median CCF values are shown (top, bars represent the median and interquartile range for each genetic alteration). Alterations with a CCF value of greater than 0.9 were defined as a clonal event. The cumulative proportion of a recurrent genetic alteration found as clonal (blue) or subclonal (red) in the cohort is shown in bottom plot. (**B**) Computational inference of temporal order of genetic alterations in MCL. Arrows indicate when paired clonal and subclonal genetic alterations were found in the same sample. Dashed lines indicate the temporal order was found in 3 or more samples; solid lines that the temporal order was found in 5 or more samples. (**C**) Kaplan-Meier plot of PFS according to the number of clonal driver events.

To understand intratumoral heterogeneity and identify the relationship of clonal and subclonal genetic events, we used the ABSOLUTE algorithm (40) to determine cancer cell fraction (CCF) for each of the genetic lesions from our 134 patients. We classified a mutation or SCNA as clonal when the CCF was greater than 0.9 and subclonal otherwise (41–43). In total, we identified 516 clonal and 173 subclonal events. Del(11q22.3), del(9p21.3), and *ATM* mutations tended to be clonal events, while mutations in *NSD2*, *PCLO*, *KMT2C*, and *LRP1B* were more likely to be subclonal events (Figure 8A; *P* < 0.05).

We further inferred temporal relationships between pairs of genetic events. We first identified instances in which a clonal event was found together with a subclonal event within the same sample, as these pairs reflected the acquisition of one lesion (clonal) followed by another (subclonal). We obtained 22 clonal and subclonal pairs and constructed a temporal map of the evolutionary trajectories of MCL based on the connections (Figure 8B). Both mutations and SCNAs could be early events (all started with IGH-*CCND1* translocation), with 6 points of departure involving mutated *ATM*, *CCND1*, del(1p), del(11q), amp(8q), and del(9q) (Figure 8B). The number of clonal events, but not of subclonal events, was associated with clinical outcomes (PFS and OS, *P* < 0.001; Figure 8C and Supplemental Figure 16, C and D), highlighting the initiating genetic events and complex genetics driving the clinical outcomes.

**Figure 9:**
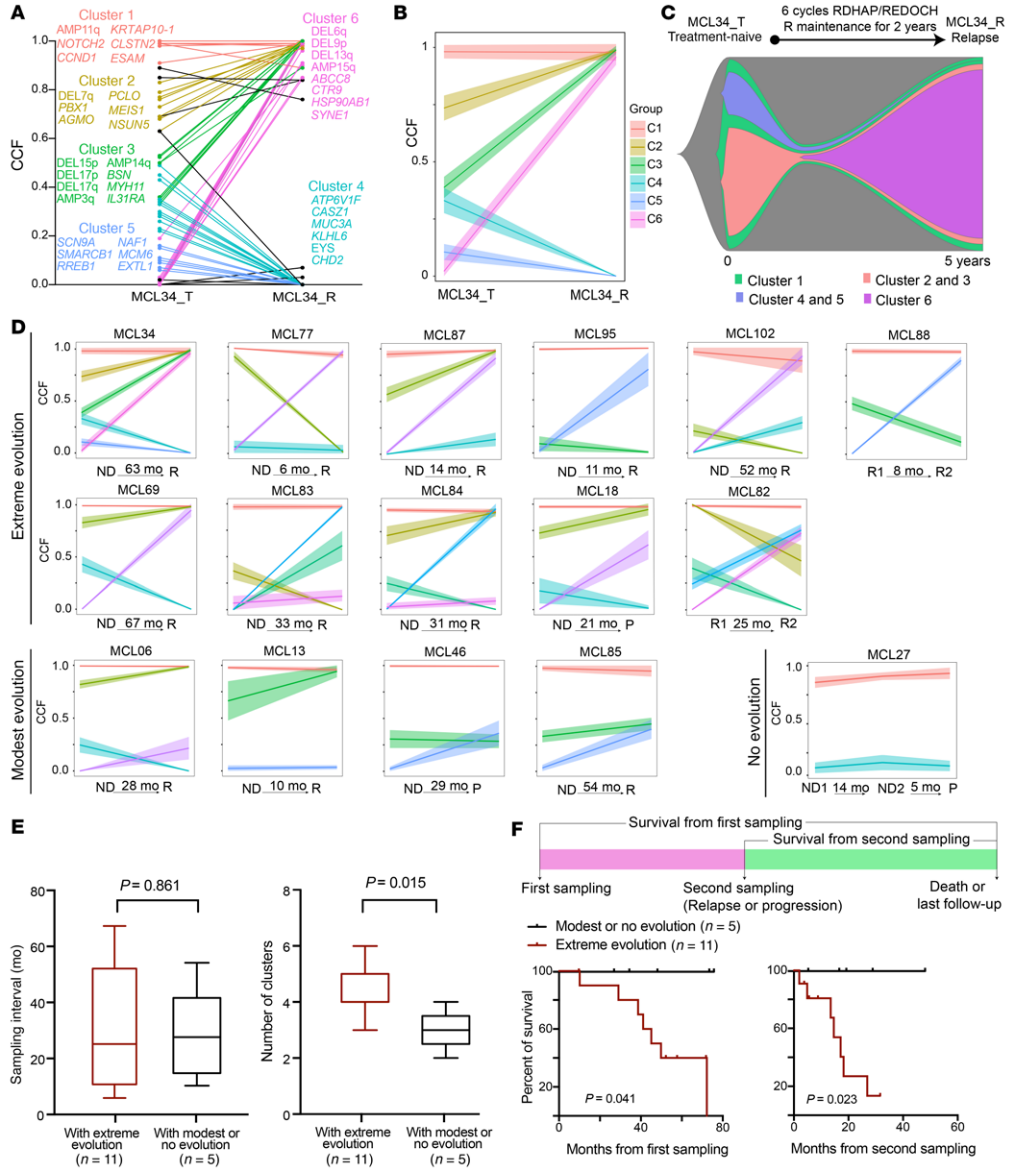
Clonal evolution pattern in MCL and its association with clinical outcome. (**A**–**C**) Depiction of tumor clonal evolution from diagnosis to relapse in a representative patient (MCL34). (**A**) Dynamic changes in genetic alterations during disease progression. Representative genetic alterations for each cluster are listed in the plot. (**B**) Clonal evolution estimated using PhylogicNDT. The mean CCF and 95% CI of each cluster are indicated. (**C**) Fish plot showing the clonal evolution process. The width of each time point indicates the clonal fractions of each subclone population. (**D**) Joint distributions of CCF values of genetic alterations across 2 (or more) time points (ND, newly diagnosed; P, progression; R, relapse; R1, first relapse; R2, second relapse) were estimated using clustering analysis. Each line corresponds to cluster of genetic alterations (range 3–33) and illustrates the dynamic changes in CCF at the different time points for clusters. We classified any CCF increase or decrease greater than 0.5 between 2 time points for any cluster as extreme evolution. CCF changes between 0.2 and 0.5 or less than 0.2 were classified as moderate evolution or no evolution, respectively. (**E**) Sample interval and number of clonal clusters in patients with either extreme evolution or with modest or no evolution. (**F**) Kaplan-Meier plot of survival from either first sampling (left) or second sampling (right).

To assess clonal evolution in relation to disease progression, we analyzed CCFs for each alteration in 33 longitudinally collected samples from 16 patients (Supplemental Figure 16A) and used PhylogicNDT to cluster dynamic changes and construct a phylogeny tree over the time points (Figure 9, A–C, Supplemental Figure 17, and ref. 44).We observed 3 patterns of tumor evolution upon treatment: (a) no clonal evolution, no change in number of clones, CCF change < 0.2 (*n* = 1 pair); (b) modest clonal evolution, 0.2 ≤ CCF change ≤ 0.5 (*n* = 4 pairs); (c) drastic clonal evolution, CCF change > 0.5 (*n* = 11 pairs) (Figure 9D). Although the time intervals between collection of first and second samples were essentially identical between drastic evolution and modest or no evolution (Figure 9E; 30.0 vs. 28.1 months, *P* = 0.861), drastic evolution showed a higher number of clusters and was significantly associated with poor survival (Figure 9, E and F, median survival from second sampling 17.1 months vs. not reached, *P* = 0.023), revealing a strong association between clonal evolution and increased disease aggressiveness. Five out of 11 patients whose samples showed drastic evolution (4 cases from C1 or C3 to C4, 1 case from C1 to C2) also had a cluster change, while all 5 patients whose samples showed modest or no evolution retained the same cluster status (Supplemental Figure 17). Patients whose samples showed cluster changes had poor survival after relapse even though their relapse interval appeared to be longer (Supplemental Figure 16, E and F), showing that genetic heterogeneity drives the progression of disease.

## Discussion

In the past decade, numerous studies have profiled genome-wide genetic alterations, gene expression, and epigenomic changes in MCL (17, 19, 21, 23, 24, 26, 31, 45–48). These studies not only generated insights into the molecular features (2, 30) and mechanisms of pathogenesis (21, 26), drug resistance (23, 24), and the cellular origin of subsets of MCL (26, 48), but also revealed the vast genetic complexity and phenotypic heterogeneity present within MCL, which has become a barrier to connecting genotype with disease phenotype in MCL. Here, starting with a large WES data set along with matched transcriptome data, we classified MCL into 4 clusters based on shared genetic lesions and determined their gene expression signatures as well as associations with clinical outcomes. We further studied clonal evolution patterns prevalent in MCL and inferred the order of genetic lesions in the development of MCL.

Our cluster analyses have a few implications. First, C1 is highly enriched for nnMCL and C2–C4 are mostly cMCL. Whereas cMCL may present with similar clinical symptoms, it fell into 3 distinct genetic subsets, which were all accompanied by coordinated dysregulated cellular pathways. This analysis provides clues for future biomarker-driven clinical trial designs that align particular treatments (e.g., Bruton’s tyrosine kinase inhibitors) with patients most likely to benefit (e.g., patients whose tumors were classified as C3 or C4 with downregulation of BCR pathway signaling). Based on the “Goldilocks” model of BCR singling and B cell survival, B cell survival is dependent on the tuning of BCR signaling such that it is neither overly strong nor overly weak (49). Within this context, cells with inherent increased BCR signaling would be anticipated to be less sensitive to a BTK inhibitor such as ibrutinib, as these cells do not meet the minimum threshold of BCR signaling needed for cell survival. In fact, MCL cell lines, including JeKo and Mino, which carry complex copy number (CN) variations and mutations (likely to be C3–C4; https://depmap.org/portal/), are sensitive to BTK inhibitor while JVM2 (genetic feature similar to C1) is insensitive to BTK treatment (50). Second, our results emphasize the influence of distinct genetic features on the clinical outcomes. Despite the different treatment regimens and patient population (Chinese and European descent) between our discovery and validation cohorts, all MCLs fell into 4 distinct clusters. These molecular clusters open a door to precision medicine, as they can serve as stepping-stones between genetic discovery and its application to clinical practice. Finally, selection of treatment for MCL based on individual genetic alteration may not be optimal because clinical response is determined by a cluster of genetic factors. *TP53* mutations are good examples illustrating this scenario, as mutant *TP53* was associated with inferior clinical courses in both previous reports (51, 52) and our analysis (Supplemental Figure 10B). In particular, C1 and C4 all harbored *TP53* mutations (36% and 63%); however, the association of mutant *TP53* and clinical outcome depends on the cooccurring genetic events (Figure 5A and Supplemental Figure 15A) and mutation sites (Figure 1, B and C, and Supplemental Figure 4B). We observed that *TP53* mutations lost their adverse prognostic significance in C1 patients, which was validated by the Barcelona cohort (Supplemental Figure 15B). This led us to consider whether the mutation sites may influence the function of TP53 protein. Based on our CLUMP analysis, some of the mutations (e.g., R273, positive Δlpvalue, enriched in SOX11-positive samples; Figure 1B) clustered together in the protein structure, while others (e.g., R248, negative Δlpvalue, more enriched in SOX11-negative samples; Supplemental Figure 4B) did not cluster that well. In support of our notion from CLUMP analysis, two recent publications reported that both R248 and R273 act in a dominant negative manner, but have different levels of impact on the function of TP53 in myeloid leukemia (53, 54), adding an extra layer of complexity for the genetic cluster. It is anticipated that integrated characterization of changes in MCL genetic clusters and gene expression following treatment with differing therapeutic interventions will further improve the design for precision medicine in MCL.

The genetic heterogeneity in MCL also has an impact on clinical outcomes and disease trajectory. As previously reported (26), we also confirmed that a high number of SCNAs is associated with inferior OS (Supplemental Figure 9F). SCNAs tend to be clonal events, and the higher number of clonal driver events is predictive of poor survival. It appears that MCL may originate from several different genetic traits, each arising from one or a combination of genetic lesions. Each trait has different intermediate and later genetic events, suggesting a stepwise acquisition of traits (Figure 8B). In nearly all MCL, t(11;14) is a foundation event, although it by itself does not lead to MCL (55). We postulate that the second hit could be a genetic trait-starting event, such as mutations in *ATM* and *CCND1*, del(11q) and del(9p) (Figure 8B). An example supporting this idea is that B cell–specific inactivation of *ATM* (one of the traits in our study) synergizes with ectopic cyclin D1 expression to promote pregerminal center lymphoma in mice (56).

Several studies reported the clonal evolution in MCL with the implication that heterogeneous genetic alterations associated with MCL relapse (8, 23, 47). Our study revealed that branched evolution is a common feature upon chemotherapy and predictive of clinical outcomes, which suggests that intratumor heterogeneity forms the fuel for relapse and drug resistance. Although mutant *TP53*/del(17p) was reported to be associated with disease relapse (25), we only observed frequency of del(9p) and amp(3q) arise in response to therapies (>20% CCF changes in 50% of samples) (Supplemental Figure 16B). Large cohorts of sample analyses are needed in order for us to fully understand the genetic events and role of clonal evolution in driving MCL.

In summary, this integrative analysis provides a framework for assessing unappreciated genetic heterogeneity in the clinically defined subtypes of MCLs and forms the basis for designing precision therapies for aggressive MCL, with genetic factors and oncogenic pathways as tractable targets.

## Methods

### Samples and genomics studies.

Diagnostic biopsy and/or blood samples representing 152 MCL tumors were obtained from 134 MCL patients. Ninety-five patients received a standard high-dose cytarabine-based aggressive regimen (Supplemental Figure 1B), while others received nonaggressive treatment (Supplemental Methods). Tumor cells were collected from bone marrow, blood, and lymph nodes (125 cryopreserved and 27 formalin-fixed, paraffin-embedded [FFPE]), with 102 (67.1%) having matched germline tissue (Supplemental Table 1). Thirty-three were longitudinal tumor samples collected from 16 patients at diagnosis (pretreatment), at progression, or at relapse following treatment (Supplemental Table 2).

WES libraries were prepared using Agilent SureSelect Human All ExonV6 (Agilent Technologies) and sequenced on the Hiseq 4000 platform (Illumina). Raw reads were aligned to the human reference genome (GRCh37/hg19) using the Burrows-Wheeler aligner (57). Somatic single nucleotide variations (SNVs) and SCNAs were called using GATK best practice somatic mutation and somatic CN variant discovery pipelines (58, 59), respectively. A tumor-only pipeline was used to filter a panel of normal samples (16,196 normal samples, Supplemental Methods; ref. 60) from the GATK4 pipeline results for samples without matched normal tissue, which yielded comparable mutation calls in paired samples (Supplemental Figure 2). MutationalPatterns (29) was used to determine de novo mutation signatures. The CLUMPS method (27) was used to assess the significance of mutational clustering in a given 3D structure. Details of the calculation of the WAP score were described previously (27). The ABSOLUTE algorithm was used to calculate the tumor purity, ploidy, and CCF for SNV and SCNA (40). Statistical methods were adapted to infer the order of genetic alterations (43). Phylogenetic analysis was performed on longitudinally collected samples using the PhylogicNDT package (61).Detailed methods are available in the Supplemental Methods.

### CRISPR library screen.

The genome-wide CRISPR library screen was carried out using the Human GeCKO v2 Library, 2-plasmid system (a gift from Feng Zhang, Broad Institute of MIT and Harvard, Cambridge, Massachusetts, USA; Addgene, 1000000049) and following the protocol as described (62). Briefly, the library contains 122,417 unique sgRNAs targeting the human genome with 6 sgRNAs per gene. The entire library, together with helper plasmids pMD2.g and psPAX2, was then transfected into HEK 293T cells, and lentiviral supernatants were collected after 2 days, followed by spin infection at 1200*g* in 2 replicates of doxycycline-inducible Cas9-expressing JeKo-1 cells for 1 hour in the presence of 8 μg/ml polybrene. Transduced cells were selected by puromycin for 3 days, and doxycycline (1 μg/ml) was added to induce Cas9 expression, followed by culturing for an additional 14 days. Genomic DNA was harvested on days 0 (day 3 in puromycin) and 14 and subjected to high-throughput sequencing to determine sgRNA abundance. MAGeCK (63) software was used to quantify sgRNA depletion or enrichment.

### Consensus clustering of genetic alterations.

All recurrent mutated genes (frequency ≥5%), IGHV mutational status, and significant regions of SCNAs (GISTIC2.0, *q* value ≤ 0.1, and frequency ≥ 10%) were assembled into a gene matrix, and NMF consensus clustering was used to identify genetic clusters as previously reported (38). Briefly, all genetic lesions were scored based on the following attributes: nonsilent mutations, 1; IGHV mutation, 2; low-level CN deletion (1.0 ≤ CN ≤ 1.7 copies), 1; high-level deletion (CN < 1.0 copies), 2; low-level amplification (2.3 ≤ CN ≤ 3.7 copies), 1; high-level amplification (CN > 3.7 copies), 2. The NMF consensus clustering algorithm was used to assign samples into different clusters. Both cophenetic coefficient and silhouette values for K = 2 to K = 10 were calculated to determine the best solution, as shown in Supplemental Figure 12A (K = 4). Fisher’s exact test was used to identify markers for each cluster by testing whether the frequency of variants in one cluster was higher than in other clusters. *P* values for multiple comparisons were adjusted using the Benjamini-Hochberg correction. Genetic alterations with *q* < 0.1 were defined as markers. The main cluster algorithm code can be accessed at GitHub (https://github.com/broadinstitute/DLBCL_Nat_Med_April_2018/tree/1c5dcd2f7b859f8b7839f4e1d9725e455b14df4d with commit ID 1c5dcd2f7b859f8b7839f4e1d9725e455b14df4d). The results were visualized as a heatmap using R package ComplexHeatmap 2.4.2 (64). In the Barcelona cohort, we adopted the single-cell projective nonnegative matrix factorization (scPNMF) (65) method to project features extracted from the discovery cohort. The parameter –K 15, method = KL was used and samples were assigned into a nearest cluster of discovery cohort based on UMAP. Thirty-three samples with matched gene expression profiling data available were used to validate our expression features in different clusters.

### Integrative genomics and transcriptomics pathway analysis.

RNA-Seq libraries were generated with the NEBNext UltraTM RNA Library Prep Kit (New England Bio) and sequenced on the HiSeq platform (Illumina). Raw reads were aligned to the human reference genome (GRCh38/hg38) using STAR (66), and expression levels of mRNAs were normalized to transcript per million (TPM). To directly compare pathway expression for each cluster, the log_2_-transformed TPM values for all genes in the gene set were averaged to provide a signature value for each sample and then the value for samples assigned to each cluster was calculated as the cluster average expression of the signature. These values were linearly transformed, and the *F* test was used to compare each cluster.

### Statistics.

Survival curves were estimated using the Kaplan-Meier method, and log-rank test was used to assess statistical significance for PFS and OS between cohorts. Multivariate Cox’s regression analysis was used to assess the independent prognostic impact from MIPI risk, IGHV mutational status, and individual genetic factors for outcomes in the MCL cohort. Student’s *t* test or Mann-Whitney *U* test was used to evaluate differences between continuous variables.

### Study approval.

All samples were obtained from MCL patients. Written, informed consent was obtained from all participants, in accordance with the Declaration of Helsinki, and the study was approved by the Institute of Hematology and Blood Disease Hospital, the Chinese Academy of Medical Sciences, and the Peking Union Medical College Ethics Committees.

## Author contributions

LW and LQ designed the study and wrote the manuscript. SY conceived the project and provided leadership. Y Yan performed experiments, analyzed data, and prepared figures. MJ performed computational analysis. SY, Y Yan, Jun Wang, FL, DZ, RC, QZ, TW, ZY, LL, WL, RL, WS, WH, WX, ZX, MH, Jianxiang Wang, and TC managed patients and collected samples. S Bhattacharya performed CLUMPS analysis. Y Wu, LY, ATP, and VNN provided CRISPR/Cas9 screen data. Y Wang, Y Yan, and Y Yu performed clinical data annotation. QS, LC, and HW were responsible for pathologic diagnosis. AFH, CL, and AD contributed to clinical data association. EC, GC, EG, and S Bea contributed to the validation Barcelona cohort. All authors reviewed the manuscript and provided final approval for submission. The order of first authors was determined by the time that each joined the project.

## Supplementary Material

Supplemental data

Supplemental table 1

Supplemental table 2

## Figures and Tables

**Table 1:**
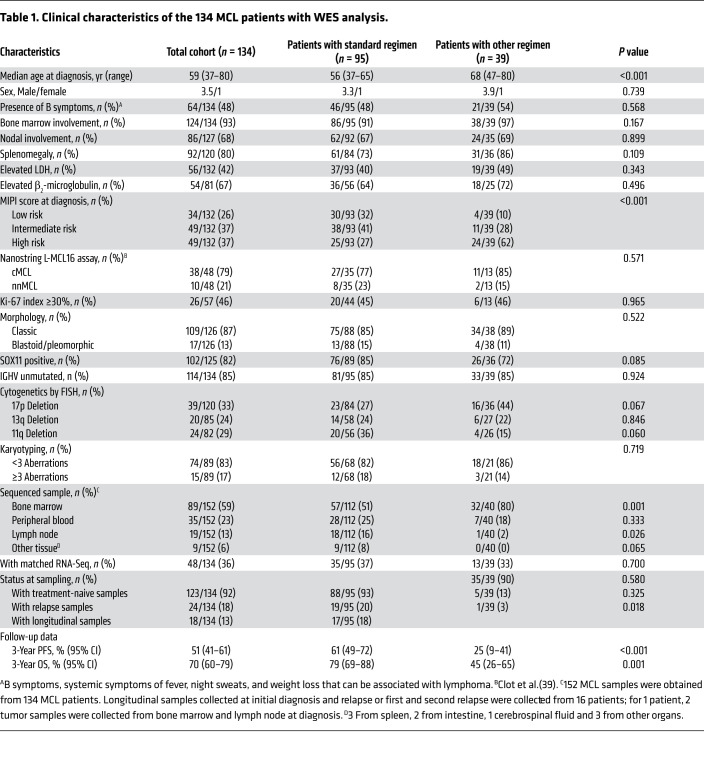
Clinical characteristics of the 134 MCL patients with WES analysis.

## Version 1 12/09/2021

In-Press Preview

## Version 2 02/01/2022

Electronic publication

## Version 3 09/29/2022

Updated supplemental data link out
