# Supplementary material for: Genomic and transcriptomic profiling reveals distinct molecular subsets associated with outcomes in mantle cell lymphoma
Source: J Clin Invest. 2022 Feb 1;132(3):e153283. doi: 10.1172/JCI153283 (PMC8803323; doi:10.1172/JCI153283)
Supplement: Supplemental data [file jci-132-153283-s011.pdf]

Supplemental Material

Genomic and Transcriptomic Profiling Reveals Distinct Molecular Subsets Associated with Outcomes in Mantle Cell Lymphoma

1. Methods

2. Supplemental Figures 1-17

Methods

Specimen and characteristics

Diagnostic biopsy and/or blood samples representing 152 Mantle cell lymphoma (MCL) tumors were obtained from 134 MCL patients with written consent at the Institute of Hematology and Blood Disease Hospital, Chinese Academy of Medical Sciences and Peking Union Medical College. One hundred and two tumor samples were with matched normal tissues (from oral mucosa or peripheral blood), which were obtained from 89 of these patients. Enrollment of patients in the BDH-MCL01 clinical trial (NCT02858804) was approved by the local Institutional Review Board. 123 patients were newly diagnosed, and therefore previously untreated, and 11 patients had relapsed. Longitudinal samples collected at initial diagnosis and relapse or first and second relapse were collected from 16 patients; for one patient, two samples were collected from bone marrow and lymph node at the diagnosis. Ninety-five of the patients (70.9%) received standard high dose cytarabine-based aggressive treatment regimen described in the prospective, non-randomized BDH-MCL01 clinical trial (NCT02858804). Patients on this clinical trial received rituximab combined with 4 cycles EDOCH (etoposide, dexamethasone, doxorubicin, cyclophosphamide and vincristine) alternating with DHAP (cisplatin, cytarabine and dexamethasone). Patients who experienced at least partial remission (PR) on this regimen underwent either upfront autologous stem cell transplant (ASCT) or two more cycles of consolidation therapy. Patients were treated with rituximab maintenance until disease progression, consent withdrawal, or unacceptable toxicity (Supplemental Figure 1B). The rest of the cohort (n=39) were treated with cyclophosphamide, doxorubicin, vincristine, prednisone (CHOP) with or without rituximab (n=15, n=10) or ibrutinib (n=14). All the 15 nnMCL patients adopt “watch-and-wait” approach. Treatment was initiated only at the time that patients become symptomatic. 10 of the 15 patients received standard cytarabine-based immuno-chemotherapy with a median time to treatment period of 9 months (1-25). Another two of the nnMCL patients received ibrutinib when there were indications for treatment. The characteristics of patients in this study are described in Table 1.

Diagnostic specimens were reviewed by a panel of three hematopathologists in accordance with World Health Organization criteria(1). All MCL samples harbored the IGH/CCND1 translocation as identified by fluorescence *in situ* hybridization (FISH). One hundred twenty-five samples (82.2%) were derived from fresh or cryopreserved peripheral blood (PB, n=35), bone marrow (BM, n=89) or cerebrospinal fluid (n=1) cells (Supplemental Figure 1A). Twenty-seven samples (17.8%) were derived from formalin fixed paraffin-embedded (FFPE) biopsy tissues; 19 from lymph nodes (LN), 3 from spleen, 2 from intestine, and 3 from other organs. Matched normal samples (n=102 [67.1%] were obtained from 89 patients. These samples were collected from oral mucosa (n=28) or PB mononuclear cells (n=61) when the patients were in complete remission or negative for minimal residual disease (sensitivity level of 10^{-4}) as evaluated by flow cytometry.

Sample processing and DNA and RNA extraction

Cryopreserved suspension tumor cells were thawed and washed with phosphate buffered saline (PBS). Tumor cells were then enriched based on CD19 expression by magnetic conjugated bead selection (Miltenyi Biotec). Purity of the enriched tumor cells was validated by flow cytometry analysis for co-expression of CD19 and CD5.

Genomic DNA was extracted using the QIAamp DNA Micro kit (Qiagen). For FFPE MCL samples, prior to DNA extraction, the slides were reviewed by hematopathologists and samples with high tumor burden were selected (>60%). DNA was extracted from FFPE samples using the QIAamp DNA FFPE Tissue kit (Qiagen). Total RNA was extracted using TRIZOL Reagent (Thermo Fisher Scientific) according to the manufacturer’s protocol. Agarose (1%) gel electrophoresis was used to assess DNA and RNA degradation and contamination. Purity of DNA and RNA was confirmed using a NanoPhotometer spectrophotometer (Implen). DNA and RNA

2

concentrations were measured using a Qubit 2.0 Flurometer (Life Technologies), and integrity was assessed using the Nano 6000 Assay Kit and a Bioanalyzer 2100 system (Agilent Technologies).

Library preparation and whole exome sequencing (WES)

Sequencing libraries were prepared using Agilent SureSelect Human All ExonV6 (Agilent Technologies) with optimized experimental procedures. Briefly, genomic DNA (1 µg per sample) was randomly fragmented to 180-280 bp using a hydrodynamic shearing system (Covaris). After fragmentation, DNA fragments were end polished, A-tailed, and ligated with the full-length adapter. Fragments with specific indexes were hybridized with biotin-labeled probes after pooling, and magnetic beads with streptomycin were used to capture the exons. Captured libraries were enriched via PCR to add index tags and prepare them for hybridization. Products were purified using the AMPure XP system (Beckman Coulter) and quantified using the Agilent Bioanalyzer 2100 system. Agilent liquid phase hybridization was used to efficiently enrich for whole exons, which were then sequenced on an Illumina Hiseq 4000 platform. Average depths for exomes of tumor cells and matched normal cells were 200x and 100x, respectively.

Alignment and quality control

The quality of the fastq sequence files exported by sequencer was checked using FastQC (http://www.bioinformatics.bbsrc.ac.uk/projects/fastqc). Adaptors and low-quality reads with lengths less than 80 bp were removed using Trimmomatic(2). The high quality paired-end reads were aligned to the human reference genome (GRCh37/hg19) using BWA with default parameters(3). Duplicate reads were removed using the MarkDuplicates program in Picard tools (http://broadinstitute.github.io/picard/). The amount of cross-individual contamination in tumor-normal pairs was estimated using GATK4 CalculateContamination, and the median contamination was estimated to be 0.3% (interquartile-range 0.1-0.4%)(4). Samples with contamination greater than 5% were filtered out. Germline mutations were called using the GATK4 HaplotypeCaller pipeline on the DNAnexus server(5). To ensure samples were paired correctly, NGSCheckMate was used to examine pairs using all detected germline mutations(6). After these quality control procedures, data for 102 matched tumor and normal tissue pairs and 50 tumor-only samples were moved forward for downstream analysis.

Somatic mutation calling

Somatic single nucleotide variations (SNVs) as well as short somatic insertions and deletions (indels) were called and filtered over 6 steps according to the GATK4 best-practices pipeline(5). 1) Base quality scores were corrected by using base quality score recalibration. 2) An in-house panel of normal derived from 128 Chinese WES of buccal swab DNA was generated using tumor-only mode of Mutect2. 3) SNVs and short somatic indels were called by using Mutect2. 4) Cross-sample contamination was estimated. 5) Orientation bias artifacts were estimated by LearnReadOrientationModel. 6) Somatic mutations arising from error and bias caused by alignment artifacts, strand and orientation bias artifacts, polymerase slippage artifacts, germline risk, and possible contamination were filtered out using FilterMutectCalls. Annotation of SNVs was performed using ANNOVAR(7) based on public database 1,000 Genomes(8), dbSNP147(9), ExAC(10), COSMICv86(11) and functional prediction resources dbNSFPv3(12). Mutations listed or have an allele frequency more than 1% in 1,000 Genomes(8), dbSNP147(9), or ExAC(10) and listed in in-house panel of normal files are filtered. All remaining recurrent non-silent mutations and mutations identified were confirmed by manual review using the Integrated Genome Viewer(13). The mutational landscape and mutation lollipop plots were generated using the R package Maftools(14) and Complexheatmap(15).

For tumor-only samples, we used tumor-only pipeline to identify genetic lesions. Basically, this pipeline was adapted from pipeline we used for tumor-normal paired samples described above by running tumor-only mode of Mutect2. To confidently identify somatic mutations in tumor-only samples, the following panel of normal

datasets was applied to filter out germline risk mutation sites. This includes: 1)a panel of normal files from Novogene that was generated from 2800 WES data and 500 WGS from a Chinese population (provided by Novogene); 2) a panel of normal files from the ChinaMap database(16), which was created using 10,588 WGS of Chinese individuals. 3) a panel of normal files derived from 1800 WES and 380 WGS (provided by St. Jude Children's Research Hospital).

Driver mutations were defined using MutSig2CV software(17). Genes with q value ≤ 0.1 and frequency > 5% were defined as driver mutations(13-15). Recurrently mutated genes are identified mutation occurred in >5 samples and with the mutation frequency >3%. To confirm the efficiency of the germline filter step, data for the 102 tumor samples that had matched normal data were reanalyzed using the tumor-only pipeline. After panel of normal filtration, the recurrent mutation landscape of matched normal samples analyzed as tumor-only was comparable with that of somatic mutations identified from normal pipeline with matched normal (Supplemental Figure 2C). The q values of candidate driver genes identified using either of the two Mutsig2CV modes were highly correlated (R^2=0.887, P=2.31×10^{-5}, supplemental Figure 2D). Fisher's exact test was used to estimate occurrence bias between tumor-only mode and matched-normal mode for all SNVs present in more than 3 samples. The expected P values were calculated using uniform distribution (Supplemental Figure 2E).

Copy number analysis from WES data
Somatic copy number alterations (SCNAs) and somatic allelic copy number variations were detected using best practices for the GATK4 somatic copy number variant discovery pipeline(4). First, interval files were preprocessed and annotated. Second, the coverage across intervals was calculated in tumor and normal samples. Third, a panel of normal coverage files was generated using the 102 normal samples from matched tumor-normal pairs. Fourth, segment copy ratio and allelic segment copy ratios were calculated and normalized. To generate an allelic copy number variation file that could work with ABSOLUTE, a WDL file from github was used (https://github.com/broadinstitute/gatk/blob/master/scripts/unsupported/combine_tracks_postprocessing_cnv).

Significant CNA regions in the cohort were detected by using GISTIC2(18). In brief, GISTIC2 assigns a G-score that considers the amplitude of the aberration as well as the frequency of its occurrence across samples for each aberration. Ninety-nine percent was used as the confidence level to determine wide peak boundaries and q value ≤ 0.1 was used to determine significantly altered regions.

To evaluate the consistence of copy number detection of tumor-only samples with samples have paired normal, the 102 matched normal samples were re-analyzed using tumor-only mode. Allelic SCNAs identified using tumor-only mode showed high correlation with those identified using matched-normal mode (Supplemental Figure 7A). In addition, the significantly deleted and amplified regions were highly consistent between the modes (Supplemental Figure 7D).

Mutual exclusivity and co-occurrence estimation
Odds ratios (OR) were calculated to assess associations among different genetic lesions; Fisher's exact test was used to determine significance. Multiple testing correction was performed using the Benjamini and Hochberg method and a false-discovery rate of 5% (q ≤ 0.05) was defined as the threshold for significance.

Estimation of purity, ploidy, and cancer cell fraction (CCF)
The ABSOLUTE algorithm was used to calculate the tumor purity, ploidy, and cancer cell fraction for somatic mutations and copy number variations(19). Candidate models were reviewed by three independent reviewers. For tumor samples that had matched normal samples, ABSOLUTE was run independently in both matched-normal and tumor-only modes. The resulting estimates of ploidy and purity showed high similarity between both

modes (Supplemental Figure 7B-C). Fluorescence *in situ* hybridization (FISH) was used to independently validate the purity of samples. Purity as determined using ABSOLUTE was highly consistent with purity estimated by FISH (Supplemental Figure 6C).

Inference of the order of genetic alterations
Statistical methods were adapted to infer the order of genetic alterations, as previously reported(20). Briefly, CCFs of mutation and SCNA pairs were first calculated for each sample in the full 134 MCL sample cohort. Then samples with paired clonal and subclonal genetic events were identified. All known driver pairs for which at least 3 clonal-subclonal orderings were observed were considered. Next, a one-sided Fisher's exact test was performed to test whether an event was more frequently changed from clonal evolution than random occurrence. Only mutations and SCNAs that occurred at a frequency > 5% were considered.

Clonal evolution of longitudinally collected samples
Phylogenetic analysis was performed on longitudinally collected samples (collected at diagnosis and at relapse or first and second relapse) using the PhylogicNDT package(21). Briefly, raw CCF probability density distributions of the somatic genetic variants identified using the ABSOLUTE pipeline were used for multi-dimensional nonparametric Dirichlet process to define the underlying clonal structure. The assignment of mutations to clusters were sampled and learned via a Markov Chain Monte Carlo (MCMC) Gibbs Sampler through a multinomial distribution. Tumor clone(s) with a CCF change between the two time points > 0.5 were defined as "extreme evolution"; a change between 0.2 and 0.5 defined as "modest evolution"; and a change <0.2 defined as "no evolution".

Mutation signature analysis
MutationalPattern was used to determine *de novo* mutation signatures(22). Briefly, all SNVs were classified into 96 trinucleotide changes in each sample. SNVs were categorized into two groups "Cluster" and "Noncluster" according to the nearest mutation distance, using the threshold of 1Kb(23). Then, "Cluster" and "Noncluster" SNVs were split in each sample to generate a 96x2M (M is the sample number) matrix. MutationalPattern was applied to discover mutation signatures by using non-negative matrix factorization (NMF) to reduce the dimensions. Cosine similarity was calculated to determine whether "extracted_signatures" were in line with the COSMIC V3 mutational signature database(24) (https://cancer.sanger.ac.uk/cosmic/signatures). The signature from COSMIC V3 with highest cosine similarity were choosed (Supplemental Figure 5B). The contribution of each mutation signature in each sample was plotted using the "plot_contribution" function in both "absolute" and "relative" modes.

CLUMPS analysis

The details of the calculation of the weighted average proximity (WAP) score are described previously(25). In brief, protein structures corresponding to each gene were downloaded from the PDB databank and the ones containing the majority of the mutated residues were used for WAP score calculation according to the following equation(25). $WAP = \sum n_q n_r e^{-\frac{d_{q,r}^2}{2t^2}}$. Here q and r are distinct protein residues, $d_{q,r}$ is the distance in Å between the centroids of the two residues, and n_q (and n_r) are sigmoidal functions: $n_q = \frac{N_q^m}{\theta_m + N_q^m}$. N_q is the number of patient samples where mutation q was observed, θ and m are constants with values 2 and 3, respectively. The significance of a given WAP score was calculated by estimating the P value from the null distribution. The null distribution was constructed by randomly selecting the residue positions from all over the protein structure and calculating the WAP scores, repeating the procedure 10,000 times. The following protein structures were used for calculating the WAP scores; ATM: (6K9L, 6K9K(26), 5NP1(27)), P53: (4IBY(28), 3IGK(29), 2BIQ(30),

2F1X(31), 3TG5(32)), NSD2: 5LSU(33), CCND1: 2W9Z(34), SP140: 6G8R, SMARCA4: 6LTJ(35), TRAF2: (1QSC(36), 3KNV(37)), PCDH10: (6VG4, 6VFW(38)), MPDZ: 2QG1, SAMHD1: 4MZ7(39).

RNA sequencing library preparation

Sequencing libraries were generated using the NEBNext UltraTM RNA Library Prep Kit for Illumina (NEB). Briefly, mRNA was purified from 1 µg of total RNA using poly-T oligo-attached magnetic beads. Fragmentation was carried out using divalent cations in NEBNext First Strand Synthesis Reaction Buffer (5X). First strand cDNA synthesis was performed using random hexamer primer and M-MuLV reverse transcriptase (RNase H-). Second strand cDNA was subsequently synthesized using DNA polymerase I and RNase H. Remaining overhangs were converted into blunt ends via exonuclease/polymerase activities. After adenylation of the 3' ends of DNA fragments, NEBNext adaptors with hairpin loop structures were ligated to the fragments to prepare them for hybridization. The library fragments were purified using the AMPure XP system (Beckman Coulter) to select fragments of 250-300 bp in length. Library quality and size was assessed using an Agilent Bioanalyzer 2100 system (Agilent Technologies).

Integrative analysis of gene expression and copy number variations

All the libraries were sequenced on Illumina Hiseq platform and 150bp paired-end 50 million reads were generated per library. Adaptors and low-quality bases were first removed from the sequencing reads using Trimmomatic(2). The remaining reads were then aligned to the human reference genome (GRCh38/hg38) using STAR with default parameters(40). Expression levels of mRNAs were normalized to transcript per million (TPM). To identify gene expression associated with SCNAs, mRNAs that were expressed independent of SNCAs were filtered out by performing Pearson correlation analysis between gene expression (log2 (TPM+1)) and copy number change of corresponding genes (log2(X/2)), where X indicates copy number of the corresponding gene. Transcripts with P value < 0.1 were defined as "SCNA sensitive mRNAs". SCNA-associated RNA expression was determined by assigning MCL samples into two groups based on the presence or absence of SCNAs. Genes with fewer than 3 samples in either group were filtered out for the statistical analysis. Absolute log2 fold change > 0.5 with q value ≤ 0.05 (Mann-Whitney U test and Benjamini-Hocherg correction for multiple comparisons) was used as threshold to define significantly dysregulated mRNAs associated with SCNAs.

Gene expression enrichment analysis

Gene expression signatures in the different MCL subsets were identified using Gene Set Enrichment Analysis (GSEA) in the 48 samples that had paired WES and RNA-seq data. These studies used three databases: KEGG pathway, HALLMARK, and Signature DB databases (updated 03/09/2020, https://lymphochip.nih.gov/signaturedb/)(41) to capture expression signatures associated with different clusters. Gene sets with P < 0.05 and FDR < 0.25 were considered significantly enriched pathways.

To directly compare pathway expression for each cluster, the log2-transformed pseudo TPM values (TMP+1) for all genes in the gene set were averaged to provide a signature value for each sample. The average signature value for samples assigned to each cluster was calculated as the cluster average expression of the signature. These values were then linearly transformed and F test used to compare each cluster.

Allele-specific expression in RNA-seq

Expression of mutations was called using the GATK4 RNA-seq short variant discovery pipeline for the 48 samples with matched WES and RNA-seq data(4). Briefly, reads were mapped to the human reference genome (GRCh37/hg19) in two-pass mode with STAR to better align the reads to novel splice junctions(40). The alignment file was transformed into a DNA aligner-like format using splitNCigarReads from GATK4. Then a somatic mutation calling module, including BaseRecalibrator, Apply Recalibration and HaplotypeCaller, was

used to detect expressed somatic mutations. Variants were filtered using the VariatFilteration command. Many mutations could not be detected by RNA somatic mutation pipeline due to lack of read coverage of the base, which indicated low expression of the genes. Therefore, read coverage was calculated for each base using BEDTools(42). Only mutations covered by more than 20 reads were included in the analysis and those with more than 5 alternative reads were considered expressed mutations. Variants with |RNA_MAF – DNA_MAF|≥0.2 and a false discovery rate ≤ 0.01 (Fisher's exact test) were considered to show allele-specific expression.

FISH and IGHV mutation analyses

FISH and IGHV mutation analyses were performed on the same sample collected for WES or RNA sequencing. Briefly, fresh tumor cells were spun onto slides and fixed in 3:1 (vol/vol) methanol–acetic acid reagent. The following enumeration probes were used in the panel: chromosome 12 centromere (CEP12), 13q14.3 (LSI D13S25 and RB-1), 17p13 (LSI TP53), and 11q22 (LSI ATM) (Vysis, Abbott). Hybridization was done on separate slides for each probe and at least 200 cells were examined. The cut-off values for trisomy 12, del(11q22), del(13q14.3) and del(17p13.1) were 1.94%, 1.81%, 6.17%, and 4.13%. The IGHV-IGHD-IGHJ junctions were amplified using the IGH Somatic Hypermutation Assay v2.0 (InvivoScribe) for the full cohort of 134 MCL samples. Sequence data were analyzed using the IMGT/V-QUEST tool (http://www.imgt.org). IGHV was considered mutated when the percentage of identity to the closest germline IGHV sequence was less than 97%.

Survival and statistical analysis

The primary endpoints were progression free survival (PFS) and overall survival (OS). PFS was defined as the interval between diagnosis and first progression or date of the last follow-up. OS was defined as the interval between diagnosis and death or last follow-up. Survival curves were estimated using the Kaplan-Meier method and log-rank test was used to assess statistical significance for PFS and OS between cohorts. Multivariate Cox regression analysis was used to assess the independent prognostic impact from MIPI risk, IGHV mutational status, and individual genetic factors for outcomes in the MCL cohort. Student's t test or Mann-Whitney U tests were used to evaluate differences between continuous variables. Fisher's exact test or chi-squared tests were used to examine the significance of differences in categorical variables. Spearman correlation was used to measure the association between continuous variables. Statistical analyses were performed using SPSS Version 21.0 or R version 3.6.2. Differences were considered statistically significant when p values were less than 0.05. P values for multiple comparisons were adjusted using the Benjamini-Hochberg correction.

Quantitative Polymerase chain reaction (PCR) validation

cDNA synthesis was performed by using 5X All-In-One RT MasterMix Synthesis System (Applied Biological Materials, Vancouver, Canada) following the manufacturer's instructions. The SYBR premix Ex TaqTM assays (Takara, Dalian, China) was then utilized to amplify specific cDNA fragments in the Prism 7500 PCR system (Applied Biosystems) to evaluate the expression of selected genes, including NAA38 (17p13.3), PRPF8 (17p13.3), NUP88 (17p13.3), PIK3CA (3q21.3), NUDT1 (7p22.3), CDK2 (12q13.3), UBE2Q2 (15q25.3), CDC16 (13q33.3), MAP2K4 (17p13.3), SNRPA1 (15q25.3). All these selected genes were located in significant SCNVs. The sequences for the primers were provided below. Data were analyzed by comparative threshold cycle method using B2M as housekeeping reference gene.

Gene	Cytoband	Copy number	Forward primer sequence	Reverse primer sequence	Size
**B2M**	15q21.1	-	GTCTTTCAGCAAGGACTGGTCT	CACGGCAGGCATACTCATCT	83
**NUP88**	17p13.3	Del	GGCAGACCTGGCTTCCTAA	GCTGGTTTCTCAGCTTCGGT	88
**NAA38**	17p13.3	Del	TTCGCATGACAGATGGACG	ACTGACCCGACGGCTTGA	108
**PRPF8**	17p13.3	Del	ACCAGCCGTTGAGGGACA	TGATTAGCCAGGCGGTAGAG	103
**PIK3CA**	3q21.3	Amp	AAGAGCCCCGAGCGTTTC	CGTGGAGGCATTGTTCTGATT	100
**NUDT1**	7p22.3	Amp	CTACTGGTTTCCACTCCTGCTT	CGCTAGACCGTGTCCACCTC	111
**CDK2**	12q13.3	Amp	TACACGTTAGATTTGCCGTACC	TAAACATTGTGGCAGCAGGA	102
**UBE2Q2**	15q25.3	Amp	TTCCGCATCGTCAGTTGG	GAAGATGGATAGGATTCCGTGA	128
**CDC16**	13q33.3	Del	AGCCACAACACTTGAGAAAACC	ACAGCATAGGCAAATGACACC	143
**MAP2K4**	17p13.3	Del	GACTTCGGCATCAGTGGACA	CAGACATCAGAGCGGACATCA	134
**SNRPA1**	15q25.3	Amp	AAAAGAGCGTCAGGAAGCAG	TTTTGTCAGTTGGCAAACCAG	119

Supplemental Figure Legends

Supplemental figure 1

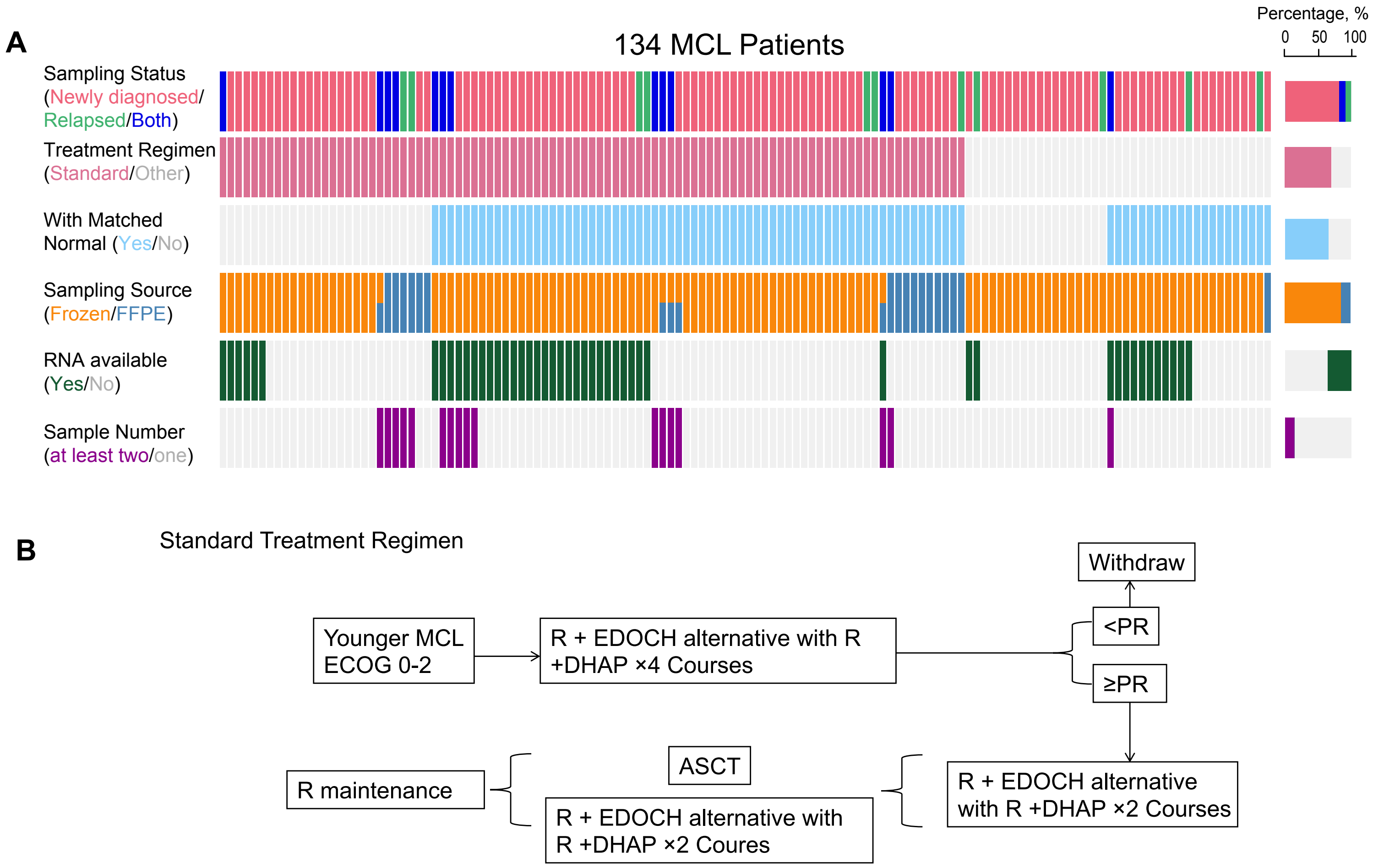

Supplemental Figure 1. Clinical sample information. A. Composition of the dataset. The dataset includes 152 samples derived from 134 MCL patients (Row 1). Ninety-five patients received a standard treatment regimen (row 2). Eighty-nine patients provided paired normal samples (102 samples) (row 3). One hundred twenty-four tumor samples were from fresh-frozen peripheral blood or bone marrow cells (row 4). Samples used for transcriptome profiling (row 5) and for clonal evolution (row 6) are noted. **B.** Schema for the treatment regimen of REDOCH/RDHAP. R, Rituximab; EDOCH (etoposide, dexamethasone, vincristine, cyclophosphamide, and doxorubicin); DHAP (dexamethasone, high dose cytarabine, cisplatin).

Supplemental figure 2

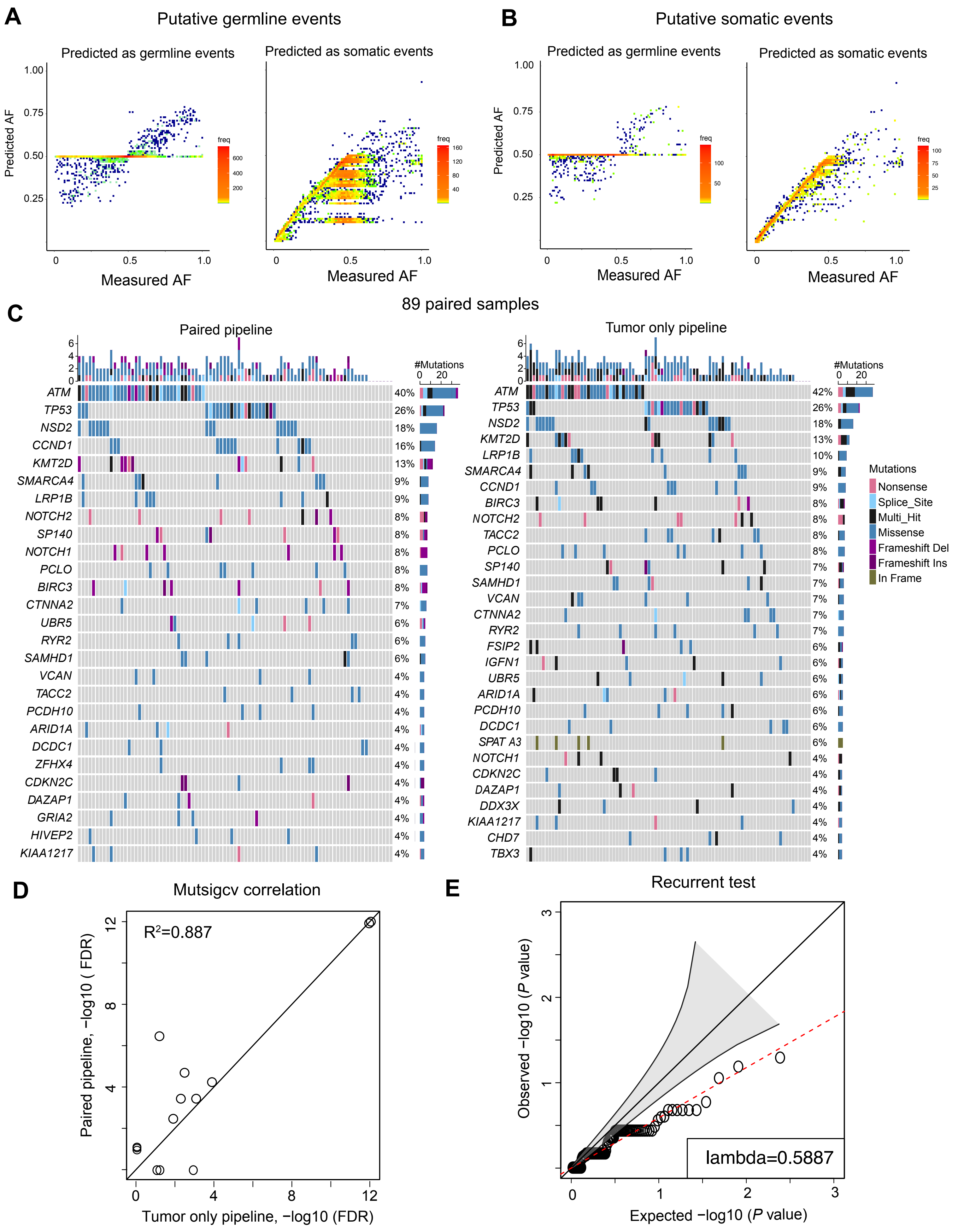

Supplemental Figure 2. Quality control for mutation call pipelines. A. Scatter plots showing the correlation between the measured allele frequency (AF) and the predicted AF for germline events identified by using the tumor-only pipeline to analyze matched-normal samples. Left: positive correlation if events are predicted as germline events by the tumor-only pipeline. Right: no correlation if the events are predicted as germline events by the tumor-only pipeline. **B.** Scatter plots showing the correlation between the measured AF and the predicted AF for somatic events identified by using the tumor-only pipeline to analyze matched-normal samples. The correlation was more pronounced when the events were predicted as somatic events than as germline events. **C.** Recurrent somatic mutations present in the 89 tumor samples that had matched normal samples. Mutations were determined using the paired pipeline (left) and the tumor-only pipeline (right). **D.** Scatter plot showing the correlation of Mutsig2CV false discovery rate (FDR) values for recurrent mutations that were called by the paired pipeline and tumor-only pipeline (events with FDR *q* value <0.1 were plotted). The plot shows the consistency of two pipelines for Mutsig2CV analyses. **E.** Two-sided Fisher's exact tests were used for somatic events determined by paired pipeline and tumor only pipeline. The observed *P* values were lower than randomly expected *P* value, indicating no significant bias of somatic mutation calls between the two pipelines.

Supplemental figure 3

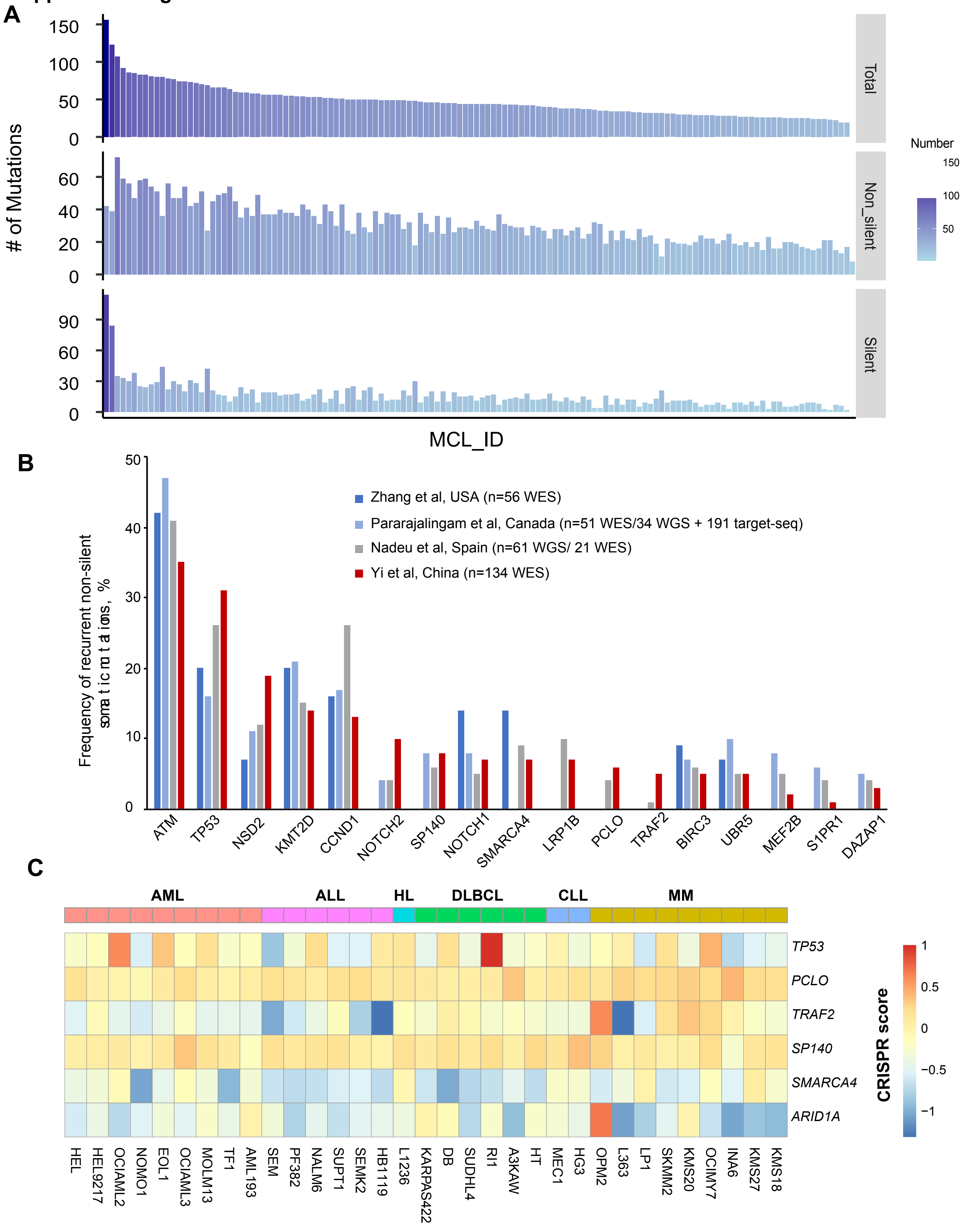

Supplemental Figure 3. Mutations in different cohorts. A. The number of non-silent and silent mutations detected in each sample from our WES analysis. **B.** Frequency of recurrent non-silent somatic mutations from 3 reported studies and our cohort. **C.** Heatmap of β scores of genes identified as having recurrent mutations genes from multiple cell lines with hematological malignancy. All the scores are from DepMap genome-wide perturbation screen.

Supplemental figure 4

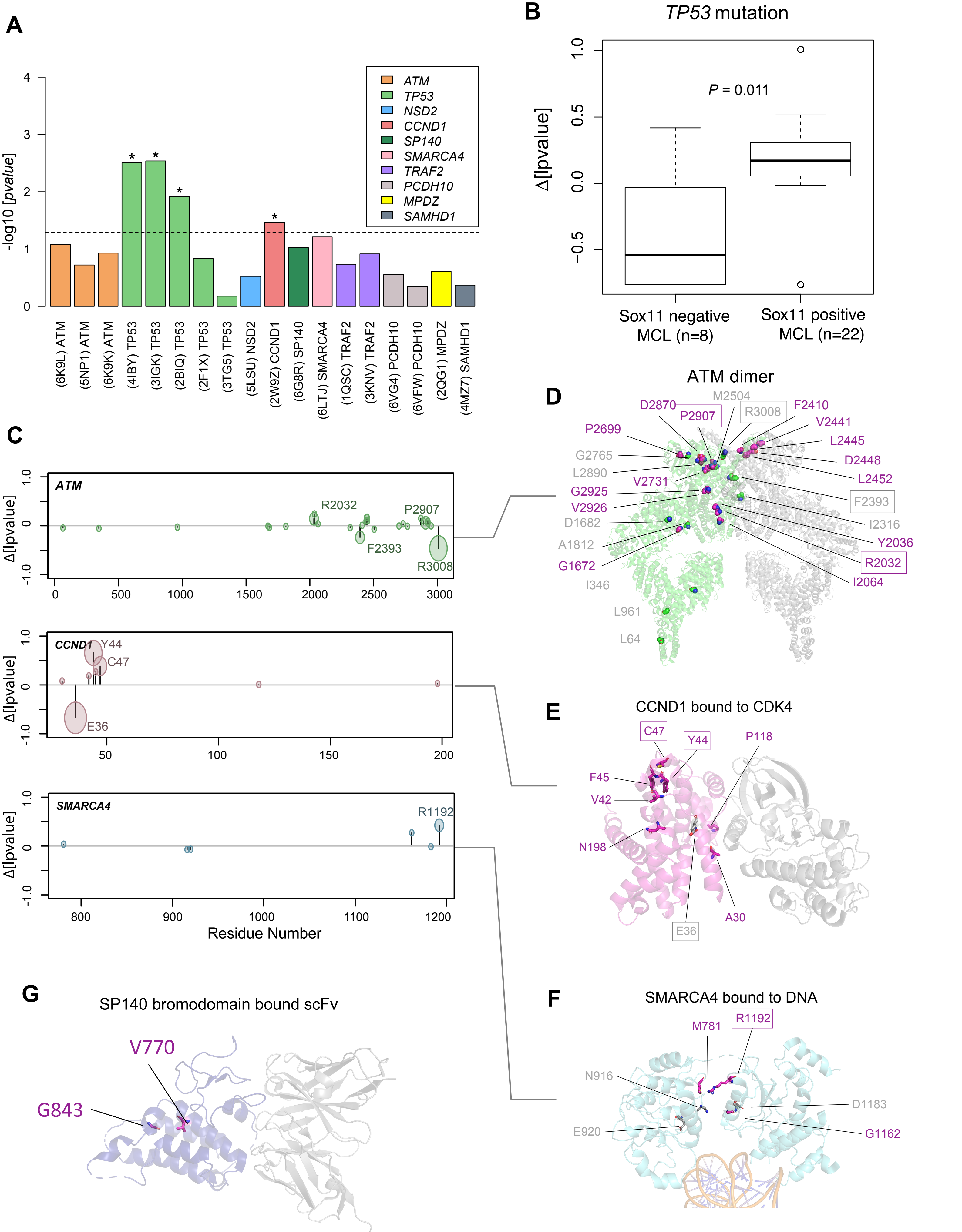

Supplemental Figure 4. Oncogenicity prediction for recurrently mutated mutations. A. Estimated *p* values calculated from the weighted average proximity (WAP) scores of protein crystal structures using the CLUMPS algorithm. Structures with significant WAP scores (*P* value < 0.05) are highlighted with asterisks. The PDB IDs of individual structures are mentioned within brackets in the labels for the bar graph. **B.** For each gene, the changes in WAP score *p* value due to removal of individual mutations are plotted as function of residue number. For mutation *i*, $\Delta[lpvalue] = log_{10}[pvalue_{all,\ no\ i}] - log_{10}[pvalue_{all\ mutations}]$. Mutations with $\Delta[lpvalue] > 0$ contribute significantly to the WAP score *p* value and therefore are expected to have high oncogenicity potential. Δlpvalue of WAP score for TP53 mutation between SOX11 negative and positive patients (student t test). **C.** Contributions of individual mutations to the collective WAP score. The radii of the circles around each point in the graphs represent the number of patients with that mutation. The *SP140* had only two missense mutations and hence the $\Delta[lpvalue]$ could not be calculated. **D-G.** Crystal structures of individual targets highlighting the oncogenic mutations. Mutations with $\Delta[lpvalue] > 0$ are colored in magenta. Mutations found in multiple patients are enclosed by boxes. **D.** ATM dimer, PDB ID: 6K9L. One of the monomer subunits is shown in green, while the other monomer is shown in grey. **E.** CCND1 bound to CDK4, PDB ID: 2W9Z. CCND1 is shown in magenta and CDK4 in grey. **F.** SMARCA4 bound to DNA, PDB ID: 6LTJ. SMARCA4 is shown in blue and DNA in orange. **G.** SP140 bromodomain bound to scFv, PDB ID: 6G8R. SP140 is shown in blue and scFv in grey.

Supplemental figure 5

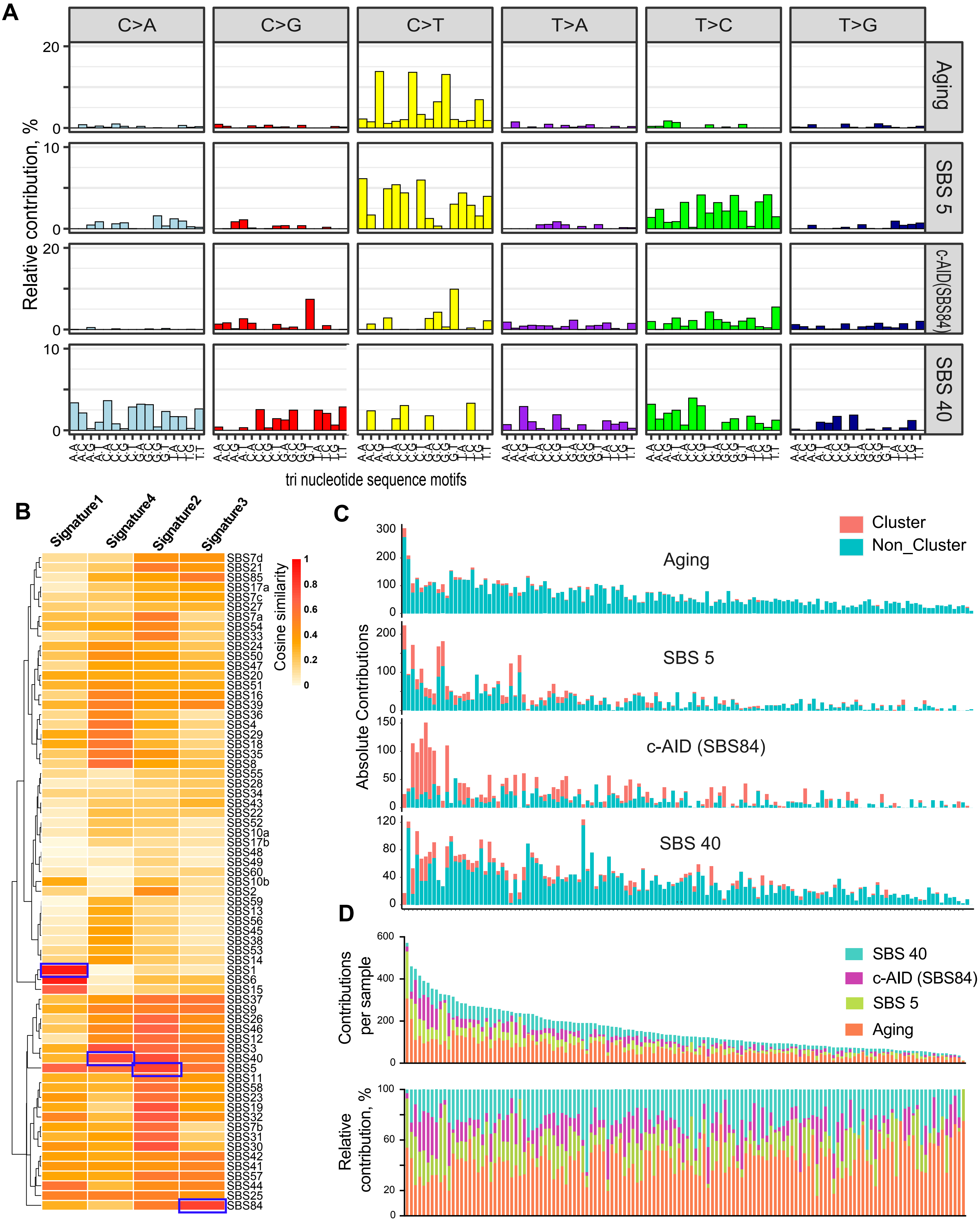

Supplemental Figure 5. **Mutational signatures in MCL. A.** The mutational signatures operating in primary MCL. Shown is the relative contribution of each indicated trinucleotide change to the four mutational signatures we identified by non-negative matrix (NMF) clustering analysis of all somatic mutations in our cohort of 134 samples. **B.** Cosine similarity between the 4 mutational signatures selected in our study and the COSMIC signatures. Blue box shows selected COSMIC signatures with highest cosine similarity to detected signatures. Signature 1 to Aging (SBS1) (cosine similarity =0.92), Signature 2 to SBS5 (cosine similarity = 0.80); Signature 3 to SBS84 (cosine similarity = 0.78); Signature 4 to SBS40 (cosine similarity = 0.76). **C.** Numbers of clustered and non-clustered mutations assigned to each mutational signature across the 134 MCL samples and sorted by decreasing mutation count. **D.** Relative enrichment of mutational signature activities in each sample.

Supplemental figure 6

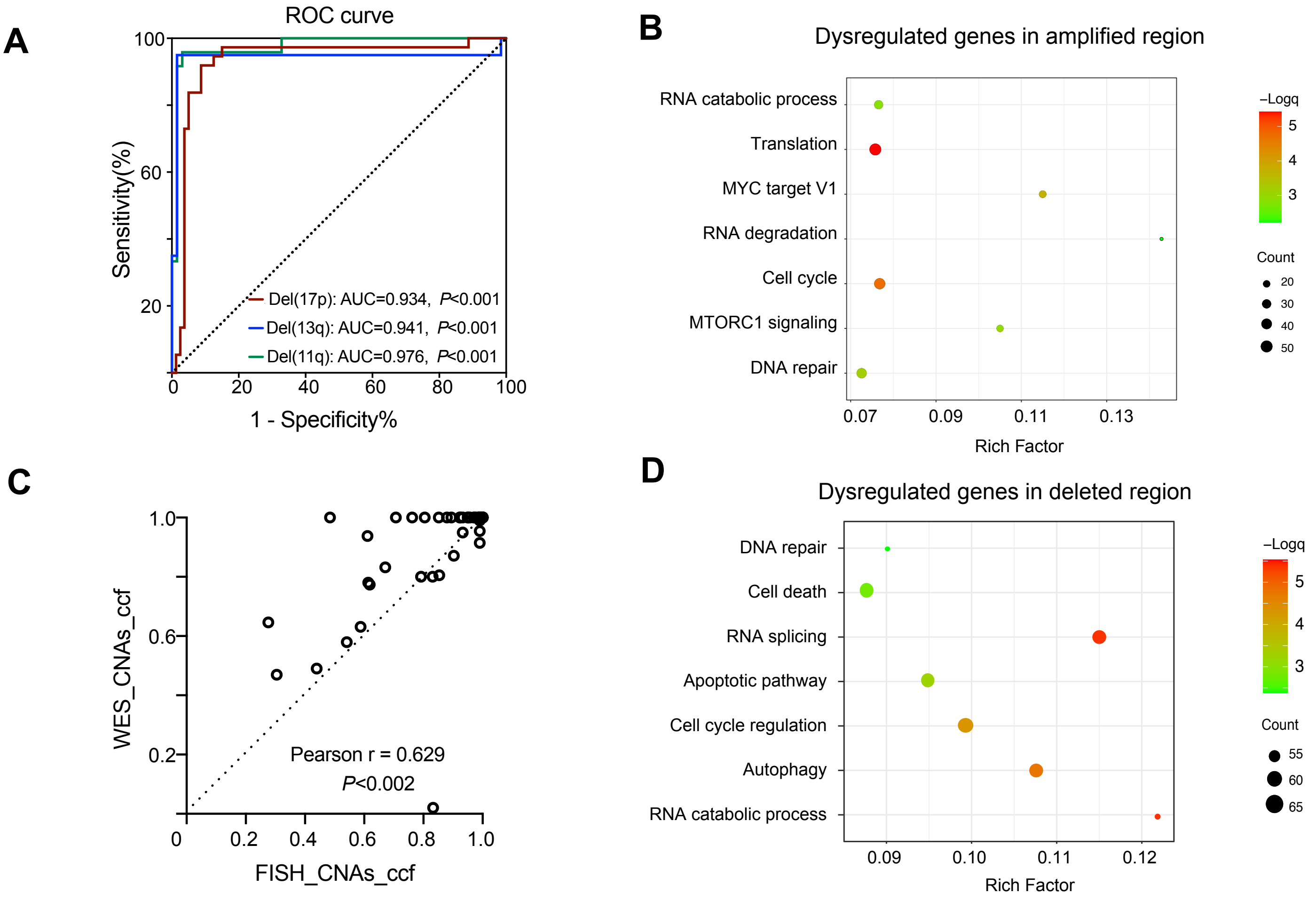

Supplemental Figure 6. Recurrent CNAs and co-occurring genetic events. A. Receiver operating characteristic (ROC) analysis was performed for the presence of del(17p), del(13q), and del(11q) based on WES data. The areas under the curves were 0.934 for del(17p), 0.941 for del(13q), and 0.976 for del(11q), indicating the accuracy of the GATK CNA pipeline compared to traditional fluorescence *in situ* hybridization (FISH). **B.** Correlation plot of CNAs called by cancer cell fraction (CCF) and FISH. **C-D.** Cellular pathway enrichment from dysregulated gene expression associated with genetic amplification (C) or deletion (D).

Supplemental figure 7

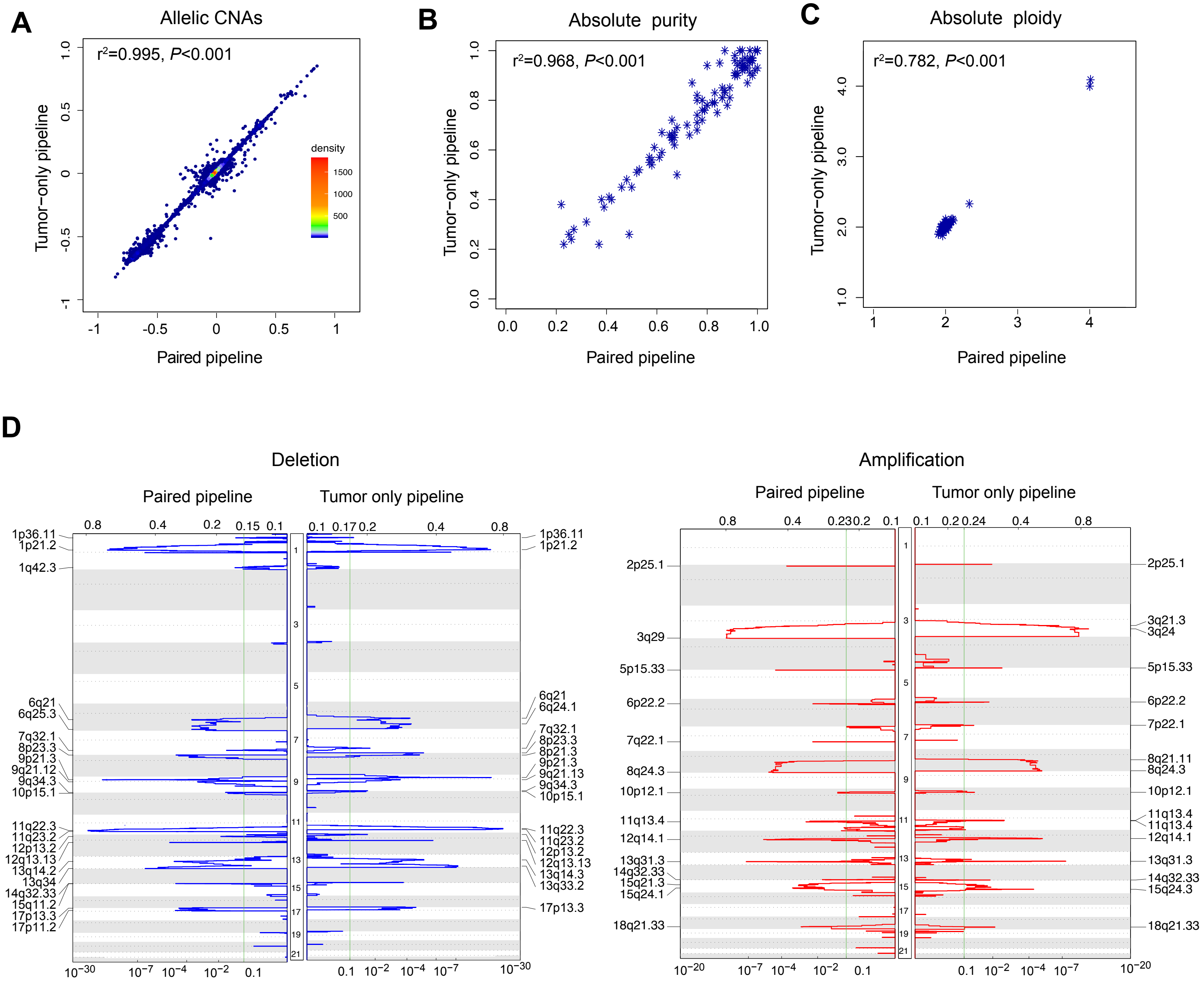

Supplemental Figure 7. **Quality control for somatic CNA pipelines. A-C.** Correlation of allelic CNAs (A), purity (B) and ploidy (C) measured using the paired pipeline and the tumor-only pipeline. **D.** Comparison of GISTIC2.0-defined significant CNAs in the 89 paired samples using the paired-pipeline (left sides of plots) and tumor-only pipeline (right sides of plots).

Supplemental figure 8

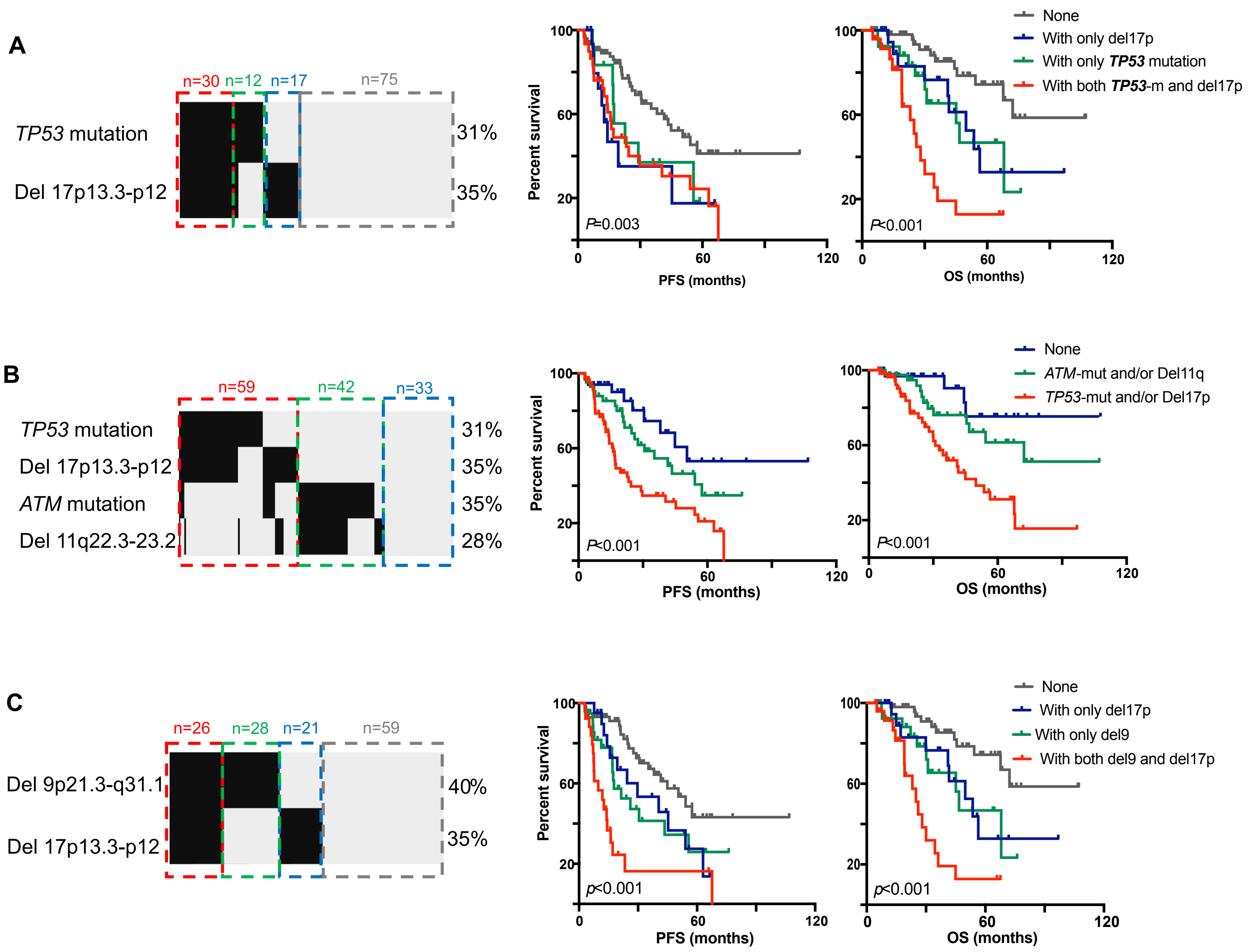

Supplemental Figure 8. Clinical impact of genetic co-occurring events in MCL. A. Kaplan-Meier plot of PFS and OS according to the co-occurrence of *del*(17p) and *TP53* mutations. Patients who had del17p-only or *TP53* mutation-only showed similar inferior PFS and OS as compared with patients who had both del17p and *TP53* mutation. **B.** Kaplan-Meier plot of PFS and OS according to the presence of *ATM* and *TP53* alteration. The two alterations show mutual exclusivity and different clinical outcomes. Patients who had *ATM* mutation had better survival than those with *TP53* mutation. **C.** Kaplan-Meier plot of PFS and OS according to the co-occurrence of *del*(17p) and *del*(9p).

Supplemental figure 9

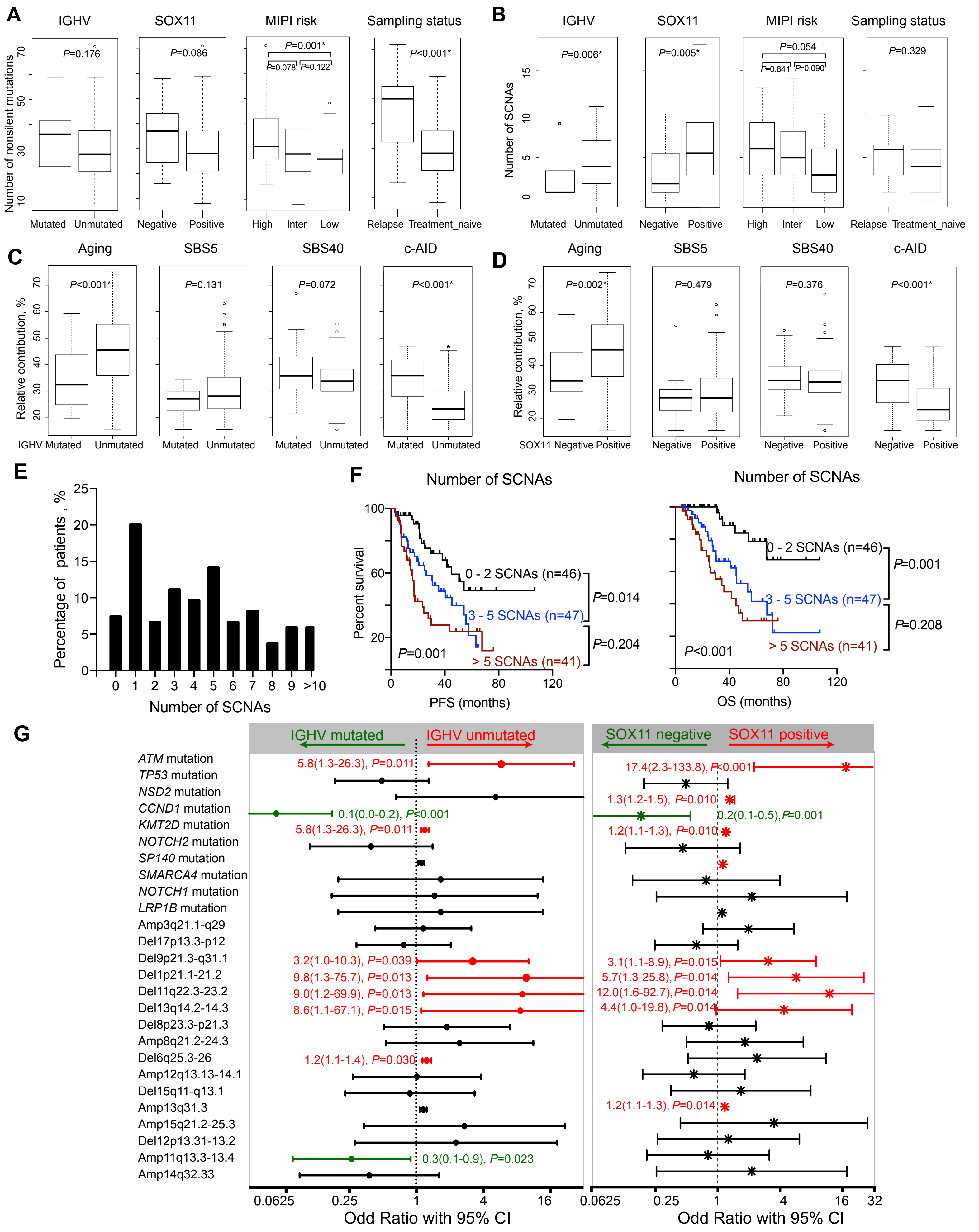

Supplemental Figure 9. Genetic alterations and their associations with clinical features. A-B. Numbers of non-silent somatic mutations (a) and SCNAs (b) and their associations with IGHV status, SOX11 expression, and MIPI risk. **C-D.** Mutation signatures and their association with IGHV mutation status (c) and SOX11 expression (d). **E**. The distribution of samples by number of SCNAs. Ninety percent of the MCL samples harbored at least one SCNA. **F.** Kaplan-Meier plots of PFS and OS according to number of SCNAs. **G.** Forest plots showing the enrichment analysis of IGHV mutation status (left panel) or SOX11 expression (right panel) with individual genetic factors. Dot represents odd ratio and bar represents 95% confidence interval (CI). Red indicates events enriched in IGHV unmuted samples or samples with SOX11 expression. Green denotes trends significantly enriched in IGHV mutated samples or samples lacking SOX11 expression (*P*<0.05).

Supplemental figure 10

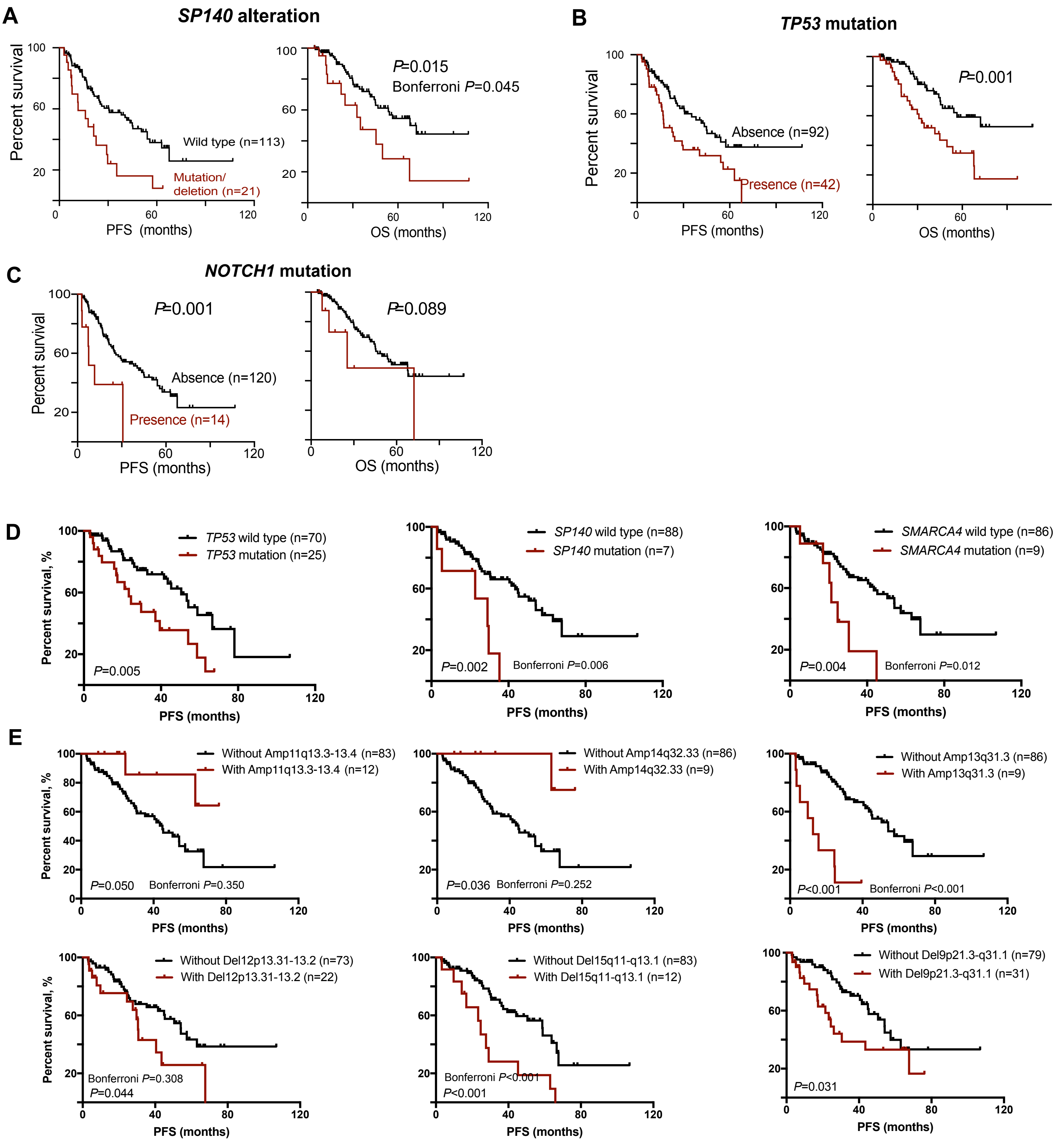

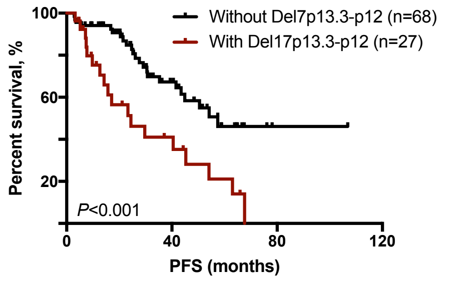

Supplemental Figure 10. Recurrent mutations associated with outcomes. A-C. Kaplan-Meier plots of PFS and OS according to presence or absence of *SP140* alteration (A) and recurrently mutated *TP53*, *NOTCH1*(B-C) in 134 MCL patients. **D-E.** Kaplan-Meier plots of PFS according to presence or absence of genetic lesions as indicated in the plots from 95 MCL patients with standard aggressive treatment regimen. For novel MCL genetic events reported in this study, a Bonferroni *P* value is also shown.

Supplemental figure 11

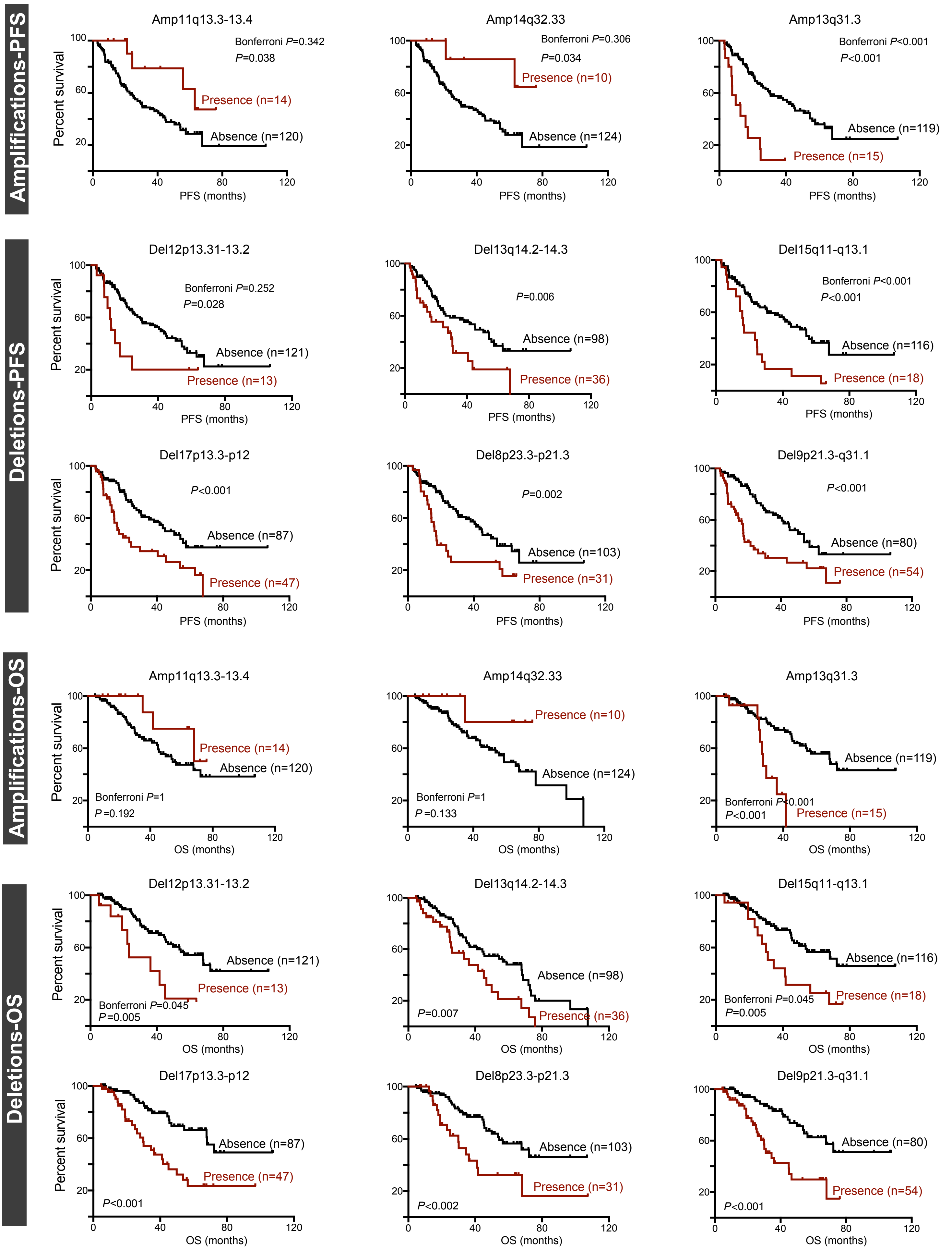

Supplemental Figure 11. Recurrent SCNAs associated with outcomes. Kaplan-Meier plots of PFS (top panel) and OS (bottom panel) according to presence or absence of recurrent SCNAs. For novel MCL genetic events reported in this study, a Bonferroni *P* value is also shown.

Supplemental figure 12

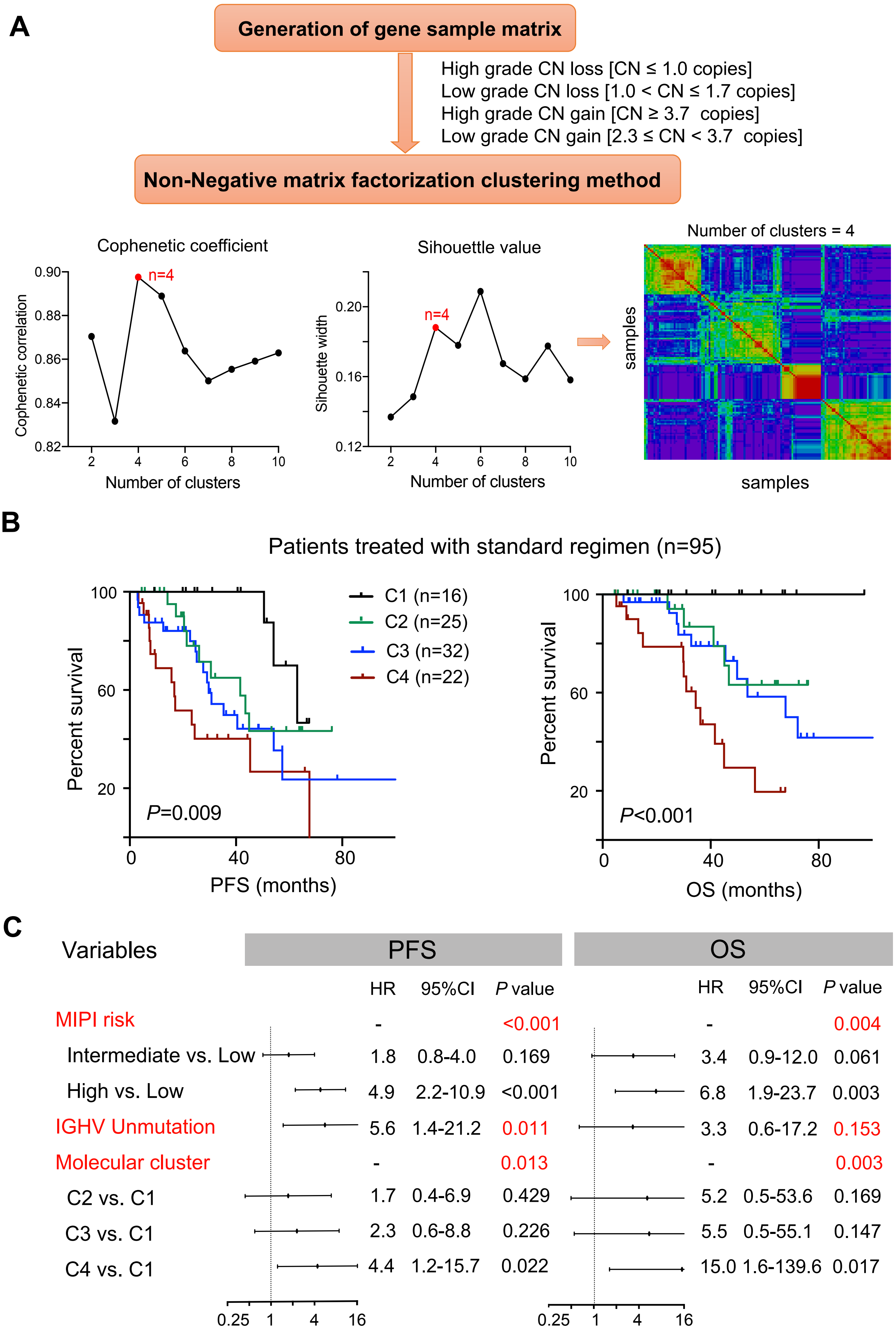

Supplemental Figure 12. NMF consensus clustering identified four genetic subtypes in MCL. A. Schema of the solution of the consensus clustering method. Plots of cophenetic coefficients and silhouette values for k=2 to k=10 cluster solutions (Bottom left). Consensus plot for k=4 cluster solutions (Bottom right). **B.** Kaplan-Meier plots of PFS and OS for the four genetic subtype clusters for the 95 patients treated with the standard regimen. **C.** Forest plots showing the multivariate analysis of MIPI risk groups, IGHV status, and genetic subtype clusters for PFS and OS in our 134 MCL cohort.

Supplemental figure 13

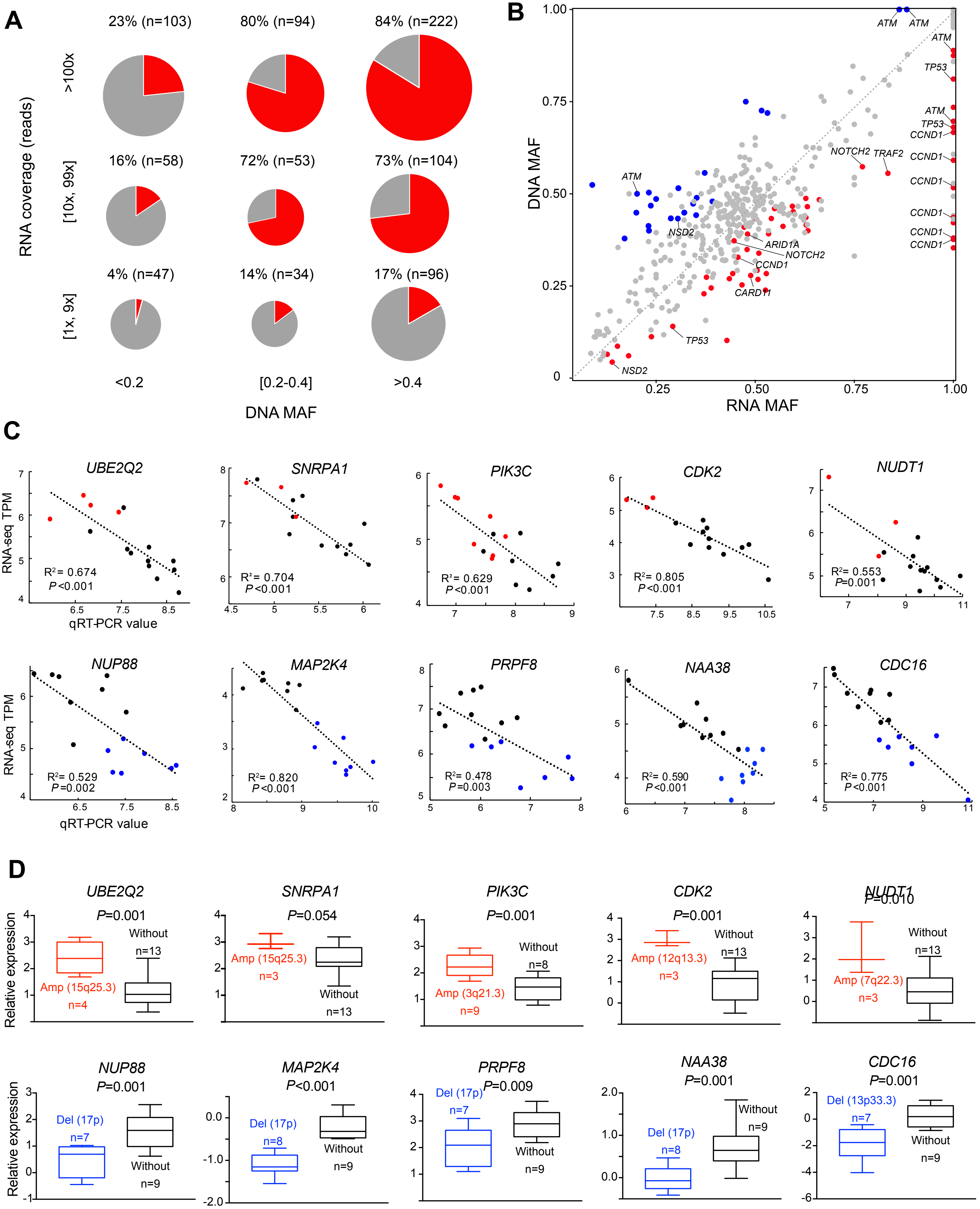

Supplemental Figure 13. Gene expression and its association with genetic subtypes in MCL. A-B. Most mutated alleles were expressed. **A.** Percentage of expressed mutations (red) categorized by DNA mutant allele fraction (MAF, x-axis) and RNA coverage (y-axis). Circle size represents mutation counts for each group. **B.** Scatter plot demonstrating the correlation between DNA and RNA MAF for expressed mutations. Red dots indicate genes that have a significant trend of mutated allele-specific expression (two-sided Fisher's exact test P <0.05 and MAF effect size >0.05); blue dots indicate genes that have a trend of elevated expression in the wild-type allele (P <0.05 and MAF effect size >0.05); grey indicates not significant. Genes with high frequency mutations and allele-specific bias expression are indicated by name. **C.** Correlation between RNA sequencing transcripts per kilobase million (TPM) and qRT-PCR. Log2 ratios of TPM are plotted in comparison with log2 of $2^{-\Delta Ct}$ values obtained from qRT-PCR. Deletions are indicated in blue and amplifications in red. **D.** Validation of gene expression changes associated with SCNAs. Relative expression (-ΔΔCt) of samples with or without CNAs was compared using student's t test. P values are displayed in the box plots.

Supplemental figure 14

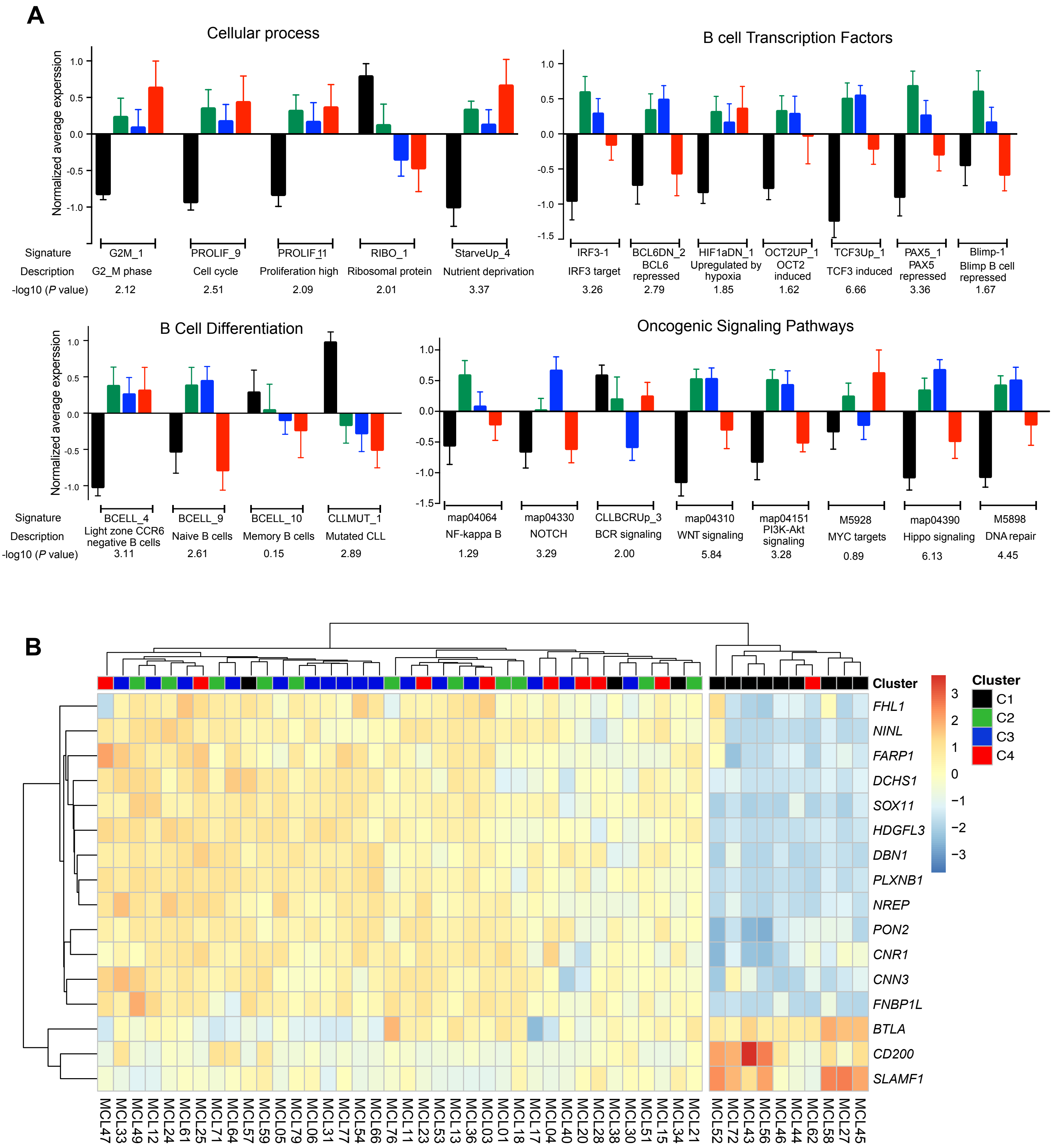

Supplemental Figure 14. Gene expression signature in different clusters. A. Bar graphs of normalized average expression for samples assigned to each cluster. Error bars denote standard error of mean (SEM). *P* values were calculated using ANOVA F-test. **B.** Validation of a 16-gene signature to distinguish cMCL and nnMCL in our cohort.

Supplemental figure 15

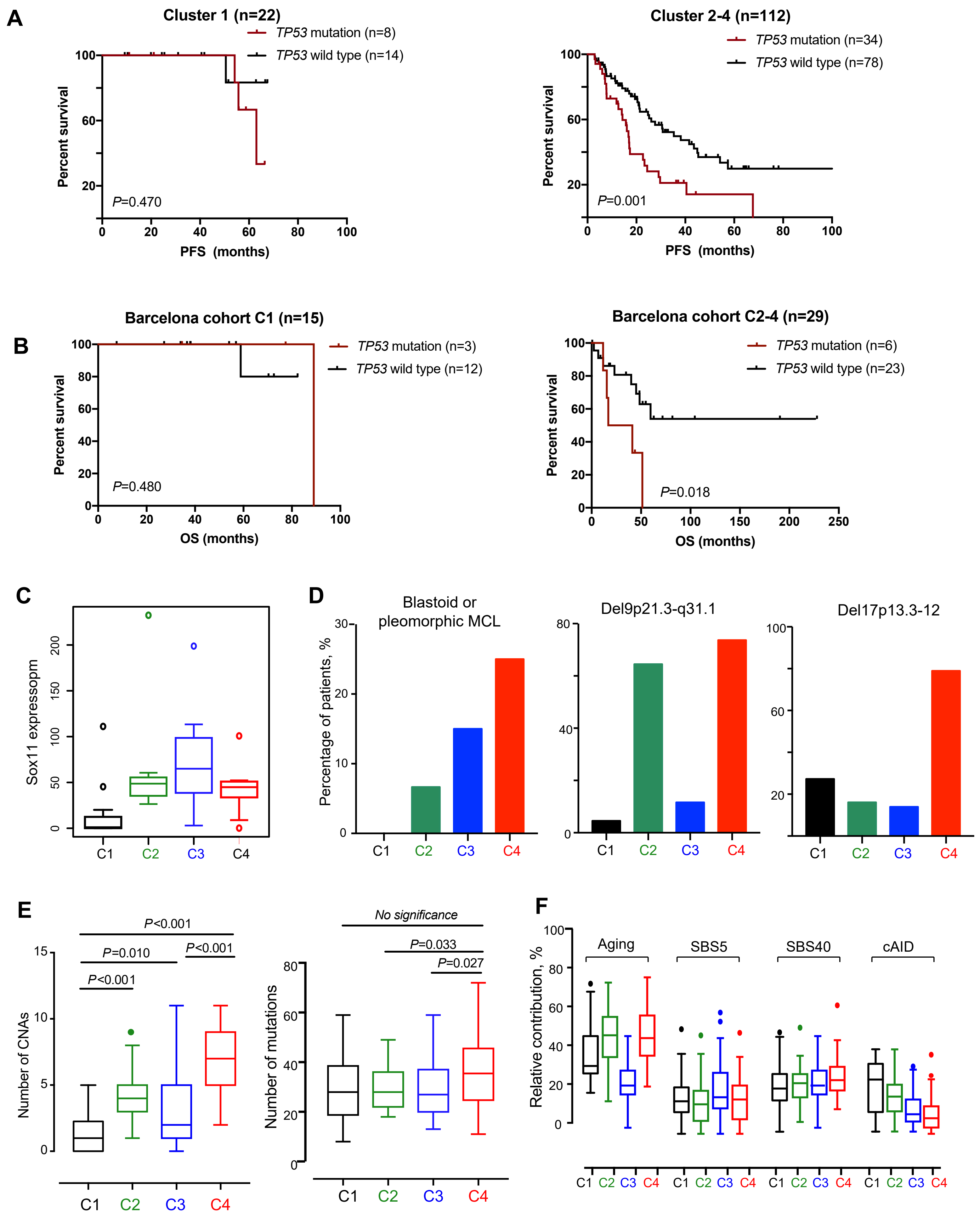

Supplemental Figure 15. Molecular features for each cluster. A-B. *TP53* mutation and their association with PFS and OS in C1 vs. C2-4 in both discovery and validation cohorts. **C.** SOX11 expression TPM value was extracted and the box-plot of SOX11 expression of the different clusters were plotted. **D-E.** Distribution of each cluster according to pathology and status of *del*(9p) or *del*(17p). Association of the genetic subtype clusters with number of SCNAs (D) and somatic mutations (E), and mutation signatures (F).

Supplemental figure 16

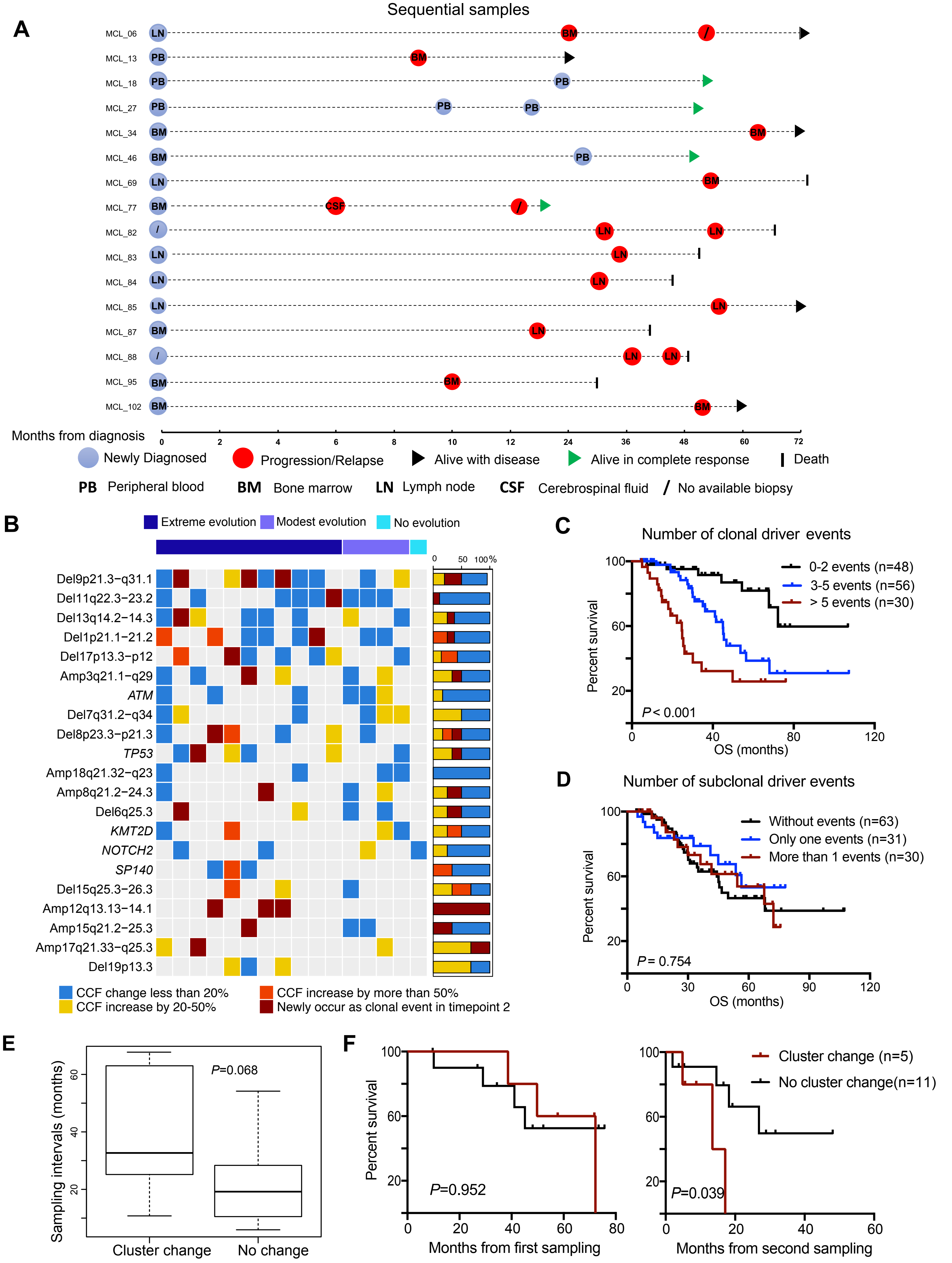

Supplemental Figure 16. Genetic lesions in the longitudinally collected MCL samples. A. Schematic of collection timepoints and tissue source for the longitudinally collected MCL samples. Samples were derived from blood (PB), bone marrow (BM), lymph node (LN), or cerebrospinal fluid (CSF) of patients at time of initial diagnosis (blue circle) and at progression or relapsed (red circle). Each row indicates an individual patient. Black triangle indicates patients was alive with disease at the time of the analysis, green triangle indicates patient was alive and in complete response at the time of the analysis, black vertical line indicates death of the patients at the indicated time. **B.** Change in cancer cell fraction (CCF) for tumor cells in samples at diagnosis and at progression/relapse. Only genetic alterations found in at least three patients are shown. Samples are grouped according to clonal evolution pattern. **C-D** Kaplan-Meier plots of OS according to the number of subclonal and clonal driver events. **E.** Sample interval in patients with and without cluster change. **F.** Kaplan-Meier plot of survival from either time of obtaining the first sample (left) or second sample (right) in patients who had or did not have a cluster change.

Supplemental figure 17-1

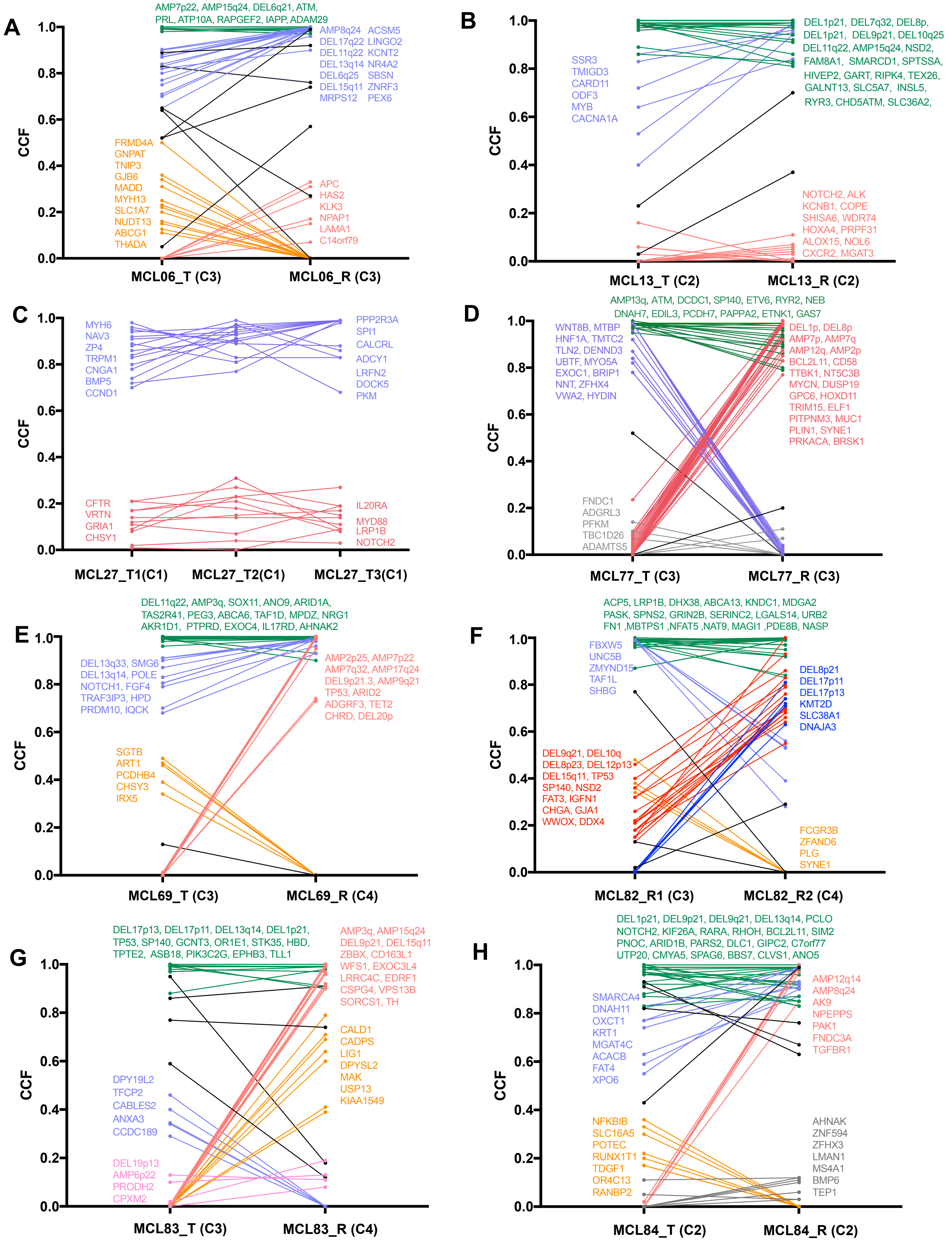

Supplemental figure 17-2

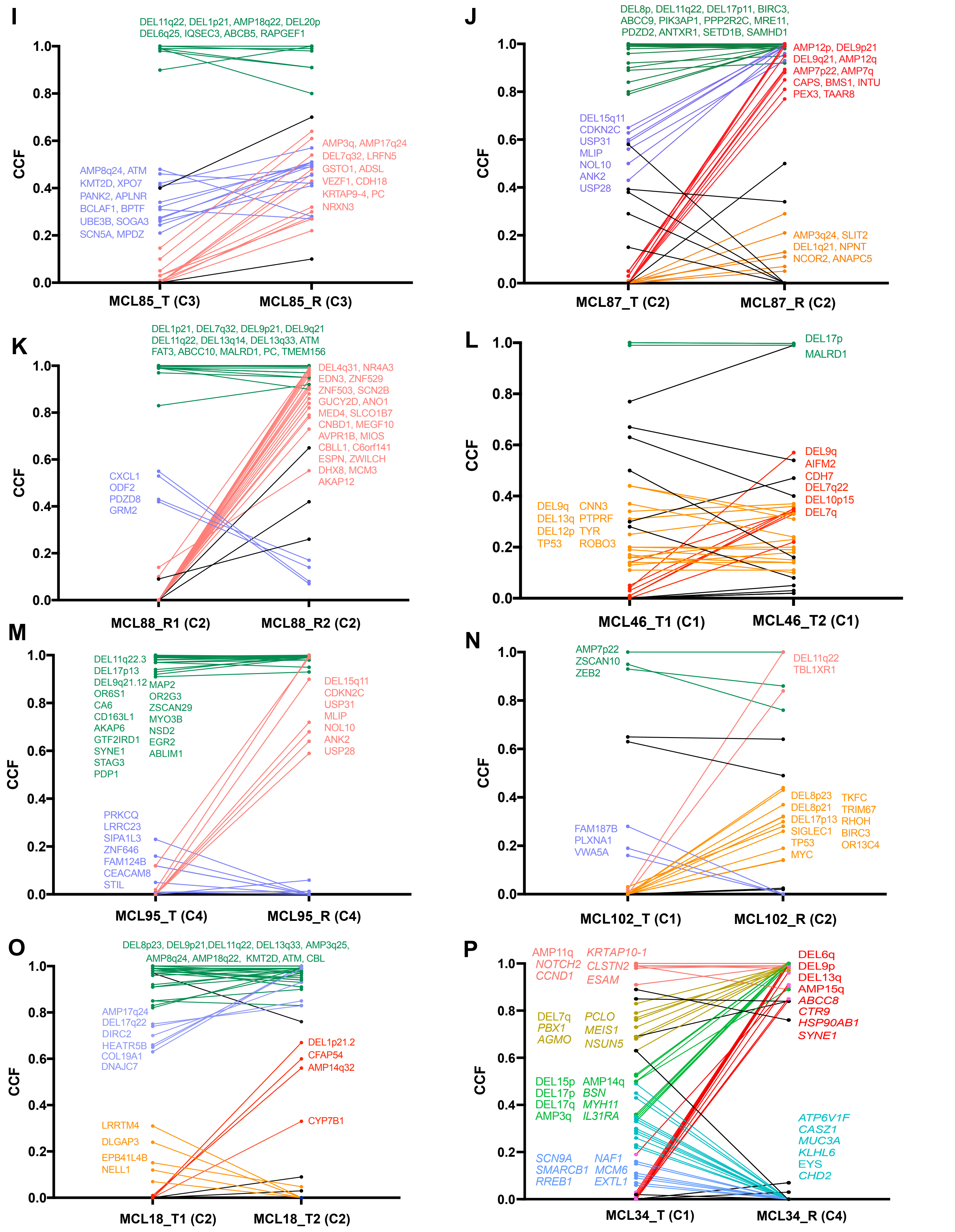

Supplemental Figure 17. Clonality changes of genetic events in our longitudinal samples. Dynamic changes in genetic alterations during disease progression from our longitudinal samples. Representative genetic alterations for each cluster are listed in the plot.

References

1. Quintanilla-Martinez L. The 2016 updated WHO classification of lymphoid neoplasias. *Hematol Oncol.* 2017;35 Suppl 1:37-45.
2. Bolger AM, Lohse M, and Usadel B. Trimmomatic: a flexible trimmer for Illumina sequence data. *Bioinformatics.* 2014;30(15):2114-20.
3. Li H, and Durbin R. Fast and accurate short read alignment with Burrows-Wheeler transform. *Bioinformatics.* 2009;25(14):1754-60.
4. McKenna A, Hanna M, Banks E, Sivachenko A, Cibulskis K, Kernytsky A, et al. The Genome Analysis Toolkit: a MapReduce framework for analyzing next-generation DNA sequencing data. *Genome Res.* 2010;20(9):1297-303.
5. DePristo MA, Banks E, Poplin R, Garimella KV, Maguire JR, Hartl C, et al. A framework for variation discovery and genotyping using next-generation DNA sequencing data. *Nat Genet.* 2011;43(5):491-8.
6. Lee S, Lee S, Ouellette S, Park WY, Lee EA, and Park PJ. NGSCheckMate: software for validating sample identity in next-generation sequencing studies within and across data types. *Nucleic Acids Res.* 2017;45(11):e103.
7. Wang K, Li M, and Hakonarson H. ANNOVAR: functional annotation of genetic variants from high-throughput sequencing data. *Nucleic Acids Res.* 2010;38(16):e164.
8. Genomes Project C, Auton A, Brooks LD, Durbin RM, Garrison EP, Kang HM, et al. A global reference for human genetic variation. *Nature.* 2015;526(7571):68-74.
9. Smigielski EM, Sirotkin K, Ward M, and Sherry ST. dbSNP: a database of single nucleotide polymorphisms. *Nucleic Acids Res.* 2000;28(1):352-5.
10. Lek M, Karczewski KJ, Minikel EV, Samocha KE, Banks E, Fennell T, et al. Analysis of protein-coding genetic variation in 60,706 humans. *Nature.* 2016;536(7616):285-91.
11. Tate JG, Bamford S, Jubb HC, Sondka Z, Beare DM, Bindal N, et al. COSMIC: the Catalogue Of Somatic Mutations In Cancer. *Nucleic Acids Res.* 2019;47(D1):D941-D7.
12. Liu X, Wu C, Li C, and Boerwinkle E. dbNSFP v3.0: A One-Stop Database of Functional Predictions and Annotations for Human Nonsynonymous and Splice-Site SNVs. *Hum Mutat.* 2016;37(3):235-41.
13. Thorvaldsdottir H, Robinson JT, and Mesirov JP. Integrative Genomics Viewer (IGV): high-performance genomics data visualization and exploration. *Brief Bioinform.* 2013;14(2):178-92.
14. Mayakonda A, Lin DC, Assenov Y, Plass C, and Koeffler HP. Maftools: efficient and comprehensive analysis of somatic variants in cancer. *Genome Res.* 2018;28(11):1747-56.
15. Gu Z, Eils R, and Schlesner M. Complex heatmaps reveal patterns and correlations in multidimensional genomic data. *Bioinformatics.* 2016;32(18):2847-9.
16. Cao Y, Li L, Xu M, Feng Z, Sun X, Lu J, et al. The ChinaMAP analytics of deep whole genome sequences in 10,588 individuals. *Cell Res.* 2020.
17. Lawrence MS, Stojanov P, Polak P, Kryukov GV, Cibulskis K, Sivachenko A, et al. Mutational heterogeneity in cancer and the search for new cancer-associated genes. *Nature.* 2013;499(7457):214-8.
18. Mermel CH, Schumacher SE, Hill B, Meyerson ML, Beroukhim R, and Getz G. GISTIC2.0 facilitates sensitive and confident localization of the targets of focal somatic copy-number alteration in human cancers. *Genome Biol.* 2011;12(4):R41.
19. Carter SL, Cibulskis K, Helman E, McKenna A, Shen H, Zack T, et al. Absolute quantification of somatic DNA alterations in human cancer. *Nat Biotechnol.* 2012;30(5):413-21.
20. Landau DA, Tausch E, Taylor-Weiner AN, Stewart C, Reiter JG, Bahlo J, et al. Mutations driving CLL and their evolution in progression and relapse. *Nature.* 2015;526(7574):525-30.
21. Leshchiner I, Livitz D, Gainor JF, Rosebrock D, Spiro O, Martinez A, et al. Comprehensive analysis of tumour initiation, spatial and temporal progression under multiple lines of treatment. *bioRxiv.* 2018:508127.
22. Blokzijl F, Janssen R, van Boxtel R, and Cuppen E. MutationalPatterns: comprehensive genome-wide analysis of mutational processes. *Genome Med.* 2018;10(1):33.

23. Chapuy B, Stewart C, Dunford AJ, Kim J, Kamburov A, Redd RA, et al. Molecular subtypes of diffuse large B cell lymphoma are associated with distinct pathogenic mechanisms and outcomes. *Nat Med.* 2018;24(5):679-90.
24. Alexandrov LB, Kim J, Haradhvala NJ, Huang MN, Tian Ng AW, Wu Y, et al. The repertoire of mutational signatures in human cancer. *Nature.* 2020;578(7793):94-101.
25. Kamburov A, Lawrence MS, Polak P, Leshchiner I, Lage K, Golub TR, et al. Comprehensive assessment of cancer missense mutation clustering in protein structures. *Proceedings of the National Academy of Sciences.* 2015;112(40):E5486-E95.
26. Xiao J, Liu M, Qi Y, Chaban Y, Gao C, Pan B, et al. Structural insights into the activation of ATM kinase. *Cell Research.* 2019;29(8):683-5.
27. Baretić D, Pollard HK, Fisher DI, Johnson CM, Santhanam B, Truman CM, et al. Structures of closed and open conformations of dimeric human ATM. *Science Advances.* 2017;3(5):e1700933.
28. Eldar A, Rozenberg H, Diskin-Posner Y, Rohs R, and Shakked Z. Structural studies of p53 inactivation by DNA-contact mutations and its rescue by suppressor mutations via alternative protein–DNA interactions. *Nucleic Acids Research.* 2013;41(18):8748-59.
29. Kitayner M, Rozenberg H, Rohs R, Suad O, Rabinovich D, Honig B, et al. Diversity in DNA recognition by p53 revealed by crystal structures with Hoogsteen base pairs. *Nature Structural & Molecular Biology.* 2010;17(4):423-9.
30. Joerger AC, Ang HC, Veprintsev DB, Blair CM, and Fersht AR. Structures of p53 cancer mutants and mechanism of rescue by second-site suppressor mutations. *J Biol Chem.* 2005;280(16):16030-7.
31. Hu M, Gu L, Li M, Jeffrey PD, Gu W, and Shi Y. Structural basis of competitive recognition of p53 and MDM2 by HAUSP/USP7: implications for the regulation of the p53-MDM2 pathway. *PLoS Biol.* 2006;4(2):e27.
32. Wang L, Li L, Zhang H, Luo X, Dai J, Zhou S, et al. Structure of human SMYD2 protein reveals the basis of p53 tumor suppressor methylation. *J Biol Chem.* 2011;286(44):38725-37.
33. Tisi D, Chiarparin E, Tamanini E, Pathuri P, Coyle JE, Hold A, et al. Structure of the Epigenetic Oncogene MMSET and Inhibition by N-Alkyl Sinefungin Derivatives. *ACS Chemical Biology.* 2016;11(11):3093-105.
34. Day PJ, Cleasby A, Tickle IJ, O'Reilly M, Coyle JE, Holding FP, et al. Crystal structure of human CDK4 in complex with a D-type cyclin. *Proceedings of the National Academy of Sciences.* 2009;106(11):4166-70.
35. He S, Wu Z, Tian Y, Yu Z, Yu J, Wang X, et al. Structure of nucleosome-bound human BAF complex. *Science.* 2020;367(6480):875-81.
36. McWhirter SM, Pullen SS, Holton JM, Crute JJ, Kehry MR, and Alber T. Crystallographic analysis of CD40 recognition and signaling by human TRAF2. *Proceedings of the National Academy of Sciences.* 1999;96(15):8408-13.
37. Yin Q, Lamothe B, Darnay BG, and Wu H. Structural Basis for the Lack of E2 Interaction in the RING Domain of TRAF2. *Biochemistry.* 2009;48(44):10558-67.
38. Harrison OJ, Brasch J, Katsamba PS, Ahlsen G, Noble AJ, Dan H, et al. Family-wide Structural and Biophysical Analysis of Binding Interactions among Non-clustered δ-Protocadherins. *Cell Reports.* 2020;30(8):2655-71.e7.
39. Zhu C, Gao W, Zhao K, Qin X, Zhang Y, Peng X, et al. Structural insight into dGTP-dependent activation of tetrameric SAMHD1 deoxynucleoside triphosphate triphosphohydrolase. *Nature Communications.* 2013;4(1):2722.
40. Dobin A, Davis CA, Schlesinger F, Drenkow J, Zaleski C, Jha S, et al. STAR: ultrafast universal RNA-seq aligner. *Bioinformatics.* 2013;29(1):15-21.
41. Shaffer AL, Wright G, Yang L, Powell J, Ngo V, Lamy L, et al. A library of gene expression signatures to illuminate normal and pathological lymphoid biology. *Immunol Rev.* 2006;210:67-85.
42. Quinlan AR, and Hall IM. BEDTools: a flexible suite of utilities for comparing genomic features. *Bioinformatics.* 2010;26(6):841-2.
